# The Efficacy of Virtual Reality-Based EEG Neurofeedback in Health-Related Symptoms Relief: A Systematic Review

**DOI:** 10.1007/s10484-025-09730-0

**Published:** 2025-07-26

**Authors:** Lina Castanho, Diogo Vicente Martinho, Ana Cristina Saial, Bruna Raquel Gouveia, Élvio Rúbio Gouveia, Filipa Ribeiro

**Affiliations:** 1 Faculty of Health Sciences and Nursing, Catholic University of Portugal, Lisbon, Portugal; 2 Laboratory for Robotics and Engineering Systems (LARSyS), Interactive Technologies Institute (ITI), Funchal, Portugal; 3 Faculty of Sport Sciences and Physical Education, University of Coimbra, Coimbra, Portugal; 4 Research Unit for Sport and Physical Activity, Coimbra, Portugal; 5 Faculty of Psychology and Educational Sciences, University of Coimbra, Coimbra, Portugal; 6 Saint Joseph of Cluny Higher School of Nursing, Funchal, Portugal; 7 Department of Physical Education and Sports, University of Madeira, Funchal, Portugal; 8 Center for Interdisciplinary Research in Health (CIIS), Lisbon, Portugal

**Keywords:** Neurofeedback, Electroencephalogram, Neuromodulation, Immersive virtual reality, Health

## Abstract

EEG Neurofeedback is a noninvasive therapeutic modality that can potentially modify targeted aspects of brain function, considered promising for the treatment of neuropsychiatric disorders. Nevertheless, some limitations have considerable potential to compromise its efficacy, including the requirement for a high number of sessions, the repetition of therapeutic tasks, and the challenge in sustaining the objectives of these tasks. These limitations can induce a decline in system dynamism and user motivation. However, barriers can be overcome by integrating immersive virtual reality into a neurofeedback system to increase motivation, interest, and adherence through immersion and a sense of presence. This systematic review aimed to determine whether this combined treatment is effective in relieving symptoms related to general health. To conduct this search following the Preferred Reporting Items for Systematic Reviews and Meta-Analyses guidelines, six electronic databases were accessed on January 26, 2024. Inclusion and exclusion criteria were defined to select the articles, and risk of bias and methodological quality assessment was conducted using the Mixed Methods Appraisal Tool and the CRED-nf checklist. A total of 24 trials were included, and according to Template for Developing Guidelines for the Evaluation of the Clinical Efficacy of Psychophysiological Interventions, the results showed that it was considered a probably efficacious treatment for attention and a possibly efficacious treatment for emotions, mood, pain, relaxation, impulsiveness, memory, self-esteem, creativity, empathy, meditation, and tiredness. Several methodological factors of the trials restrict the generalization of these findings and require caution in their interpretation, despite the promising results in future directions.

**Supplementary Information:**

The online version contains supplementary material available at 10.1007/s10484-025-09730-0.

## Introduction

Neurofeedback (NFB) is a biofeedback modality that involves the real-time monitoring and self-regulation of brain activity through sensory channels and its feedback to the participant (Sitaram et al., 2017; Weber et al., 2020). It is derived from neuromodulation therapies, the scope of which can be applied to any medical, surgical, or physiological therapy aimed at modifying nervous system function in a specific direction (Ishii et al., 2019). The NFB differs from conventional self-regulatory and cognitive behavioral techniques in that it is designed to directly target and alter brain activity (Wider et al., 2024). EEG NFB allows the brain to recognize and self-regulate its electrical activity through specific treatment procedures that either enhance (strengthen) or inhibit (weaken) certain brainwave patterns (Wider et al., 2024), inducing neuroplasticity changes (Sitaram et al., 2017). Behavioral changes resulting from self-regulation of neural activation may suggest that the physiological effects of NFB can be considered a form of endogenous neural stimulation (Sitaram et al., 2017; Sulzer et al., 2024). Thus, NFB has been used to modulate functional networks relevant to behavior (Sitaram et al., 2017). Learning to regulate the brain with NFB is a process similar to the development of skills, including the cortico-striatal circuit and its dopaminergic and glutamatergic synaptic organization (Sitaram et al., 2017).

In short, the NFB aims to adjust brainwave frequencies in certain regions of the brain associated with identified emotional/behavioral problems, as well as to improve the stability and communication of neural networks in the brain and between or within hemispheres, leading to specific behavior changes (Loriette et al., 2021; Wider et al., 2024). As a result, it is regarded as a promising approach capable of influencing both neuroscience research and clinical treatment of neuropsychiatric disorders (Hampson et al., 2020; Ishii et al., 2019).

Over the years, research has focused on evaluating the NFB efficacy, with generally promising outcomes across several health conditions, including Attention-Deficit/Hyperactivity-Disorder (ADHD) (Arns et al., 2020), Obsessive–Compulsive Disorder (OCD) (Rance et al., 2023), Depression (Patil et al., 2023; Trambaiolli et al., 2021)​, Anxiety (Chen et al., 2021; Liu et al., 2022), Post-Traumatic Stress Disorder (Askovic et al., 2023; Choi et al., 2023), Insomnia (Kwan et al., 2022), Autism (Pineda et al., 2014), physical pain, post-stroke cognitive rehabilitation (Renton et al., 2017), etc. The principles of NFB can also be applied to healthy participants to improve cognitive performance (Uslu & Vögele, 2023)​, such as memory ​(Lin et al., 2024; Zhou et al., 2024)​ and executive functions (Viviani & Vallesi, 2021). In addition, its applicability in optimizing sports performance is noteworthy (Corrado et al., 2024)​.

The efficacy of this treatment is evidenced by its ability to complement and enhance first-line treatments. For example, in Attention Deficit Hyperactivity Disorder (ADHD), NFB has shown potential, particularly given that pharmacological interventions, although effective, can be limited, cause side effects in sleep and eating, and lack solid evidence of long-term benefits (Saif & Sushkova, 2023; Shojaei et al., 2024). Additionally, a recent systematic review (Zafarmand et al., 2022) have indicated that this treatment is promising in the remission of OCD residual symptoms (after first-line treatments), reducing the duration of long and high-risk treatments, and the use of presumed interventions, including deep radiosurgery and brain stimulation (Zafarmand et al., 2022). There have been international efforts to scientifically support the efficacy of NFB (Sitaram et al., 2017), resulting in the validation of standard methodologies for reporting results (Davelaar, 2018). Despite all the benefits identified in NFB studies, it is important to note that it has limitations, including the high number of required repetitive sessions, involving computerized tasks with difficult therapeutic aims, and the difficulty of keeping patients engaged (Guedj et al., 2023). The majority of NFB systems employ visual feedback, for example in the form of two-dimensional objects that change size or move depending on changes in EEG parameters (Kober et al., 2024). One potential solution that has been proposed is the incorporation of immersive Virtual Reality (VR) into the NFB system (Guedj et al., 2023) as a factor for increasing positive affect, motivation, user experience, involvement, and focus on training (Kober et al., 2024; Lüddecke & Felnhofer, 2022). The concept of VR involves three-dimensional computer-generated simulations that provide visual, auditory, tactile, and olfactory stimuli (Emmelkamp & Meyerbröker, 2021). By replacing screen feedback (conventional NFB) with immersive 3D feedback (provided by VR), changes in brain activation can also be transferred to changes in a VR scenario, allowing more entertaining content to be adopted and treatment efficiency (Berger et al., 2022; Kober et al., 2024).

New studies have emerged in this field, showing the relevance of adapting NFB training paradigms in order to enhance its attractiveness and efficiency, using VR, as well as investigating these results in more detail in future studies [e.g., 33–35].

To the best of our knowledge, a systematic literature review regarding the efficacy of NFB and VR synergy, and its applications in healthcare, has not yet been conducted. Following on from this, the present research explores the potential applications of this combined treatment in the health field. It aims to answer the following question: *Is EEG NFB combined with (immersive) VR an effective treatment for health-related symptom relief?*

To assess risk of bias and quality, the Mixed Methods Appraisal Tool (MMAT) (Hong et al., 2018) and the CRED-nf checklist (Ros et al., 2020) were employed. The MMAT facilitates the critical appraisal process in systematic mixed-studies reviews, and the CRED-nf checklist accesses the methodological rigor and better highlights the mechanisms underlying NFB, providing a more robust, specific, and multifaceted assessment of the quality of studies (Ros et al., 2020; Viviani & Vallesi, 2021).

## Methods

This systematic review was conducted in accordance with the Preferred Reporting Items for Systematic Reviews and Meta-Analyses (PRISMA) guidelines (Page et al., 2021) and was pre-registered in the International Prospective Register of Systematic Reviews (PROSPERO), with registration number CRD42024502728.

### Eligibility Criteria

Original studies were selected for inclusion based on the following criteria, according to the PICOS structure (Amir-Behghadami & Janati, 2020):

- Population: people in any condition;
- Intervention: Neurofeedback (EEG-based) and (immersive) Virtual Reality integrated treatment;
- Comparator: Control Group (CG) (CG with only VR intervention, CG with only Neurofeedback intervention, CG with treatment as usual, placebo group/sham NFB, CG with no intervention) and pre-post comparison (i.e., no CG);
- Outcome: Health-related symptom relief;
- Study design: Studies with pre- and post-assessment.

The exclusion criteria were review studies, meta-analyses, scoping reviews, interventions that did not include VR and NFB (EEG-based) as an integrated intervention, and studies with non-health outcomes. The population was not restricted in terms of age to obtain a comprehensive view of the applicability and efficacy of this treatment. There were no restrictions on the publication dates of included articles. Only articles in English and Spanish were included in this study.

### Information Sources

A preliminary search was performed to identify terms related to NFB and VR integrated treatment. Subsequently, several electronic searches were conducted in several databases on January 26, 2024, namely PubMed, Medline Complete EBSCO Host, Cochrane Library, Psychology & Behavioral Sciences Collection, Scopus, and Web of Science.

The reference lists of the included articles were checked to identify additional articles.

### Search Strategy

Systematic literature searches using titles, abstracts, and keywords were performed using the listed databases. Keyword searches were performed using MeSH and APA terms with no date restrictions (see Supplementary Table 1 for further details).

### Selection and Data Collection Processes

Prior to the initial screening, duplicate articles were removed using the Rayyan platform (https://www.rayyan.ai/). Two researchers (L. C. and A. S.) conducted the initial screening independently on the same platform. The two researchers also independently conducted a second screening, and discrepancies were resolved through discussion until a consensus was reached. In cases in which there was no consensus, a third author (F. R.) was consulted until a decision was made. LC and AS also performed data extraction, and a consensus was reached on the final list. A data extraction spreadsheet was created to simplify this information.

### Data Items

The main outcome in this review was health-related symptom relief, as evaluated through assessment instruments at different time points, including before, after, and/or at follow-up treatment. Neurophysiological changes were defined as secondary outcome, assessed through EEG data analysis.

For each study, the following items were extracted: author and date; study design; sample size; participants’ symptoms and/or inclusion/exclusion criteria; age range; country; key findings; practical implications; outcomes; outcome measures; comparator; NFB protocol; number and frequency of sessions; and session duration (minutes).

Missing data were resolved by contacting the corresponding authors of the studies to request the information. In the absence of a response, the articles were retained as they provided relevant information to this review.

### Study Risk of Bias and Quality Assessment

The Mixed Methods Appraisal Tool (MMAT) (Hong et al., 2018), version 2018, was used to assess the risk of bias. This tool facilitates the critical appraisal process in systematic mixed-studies reviews, providing a single tool with methodological quality criteria for different study designs (Hong et al., 2018), as this review combines quantitative, qualitative, and mixed-methods studies. The MMAT tool addresses methodological criteria and includes five major quality criteria for each of the following five categories of study design: qualitative, randomized controlled and non-randomized trials, quantitative descriptive, and mixed methods (Hong et al., 2018).

The quality assessment aimed at identifying studies with more rigorous methodologies. However, it was not intended to exclude those of lower quality.

Two researchers (LC and AS) independently conducted the methodological quality assessment and resolved any discrepancies through discussion until a consensus was achieved.

The authors of the tool do not recommend the use of an overall score as this does not allow the problematic aspects of the studies to be identified. This tool is useful for qualitative and quantitative sensitivity analyses (Hong et al., 2018). The percentage of quality criteria met was calculated for each study based on the individual assessments.

In addition, for quality assessment, an evaluation was carried out using the CRED-nf checklist, a recent and specific instrument for NFB studies, which arose from the need to support methodological rigor and better highlights the mechanisms underlying NFB (Ros et al., 2020; Viviani & Vallesi, 2021). The purpose of this checklist was to obtain a more robust, specific, and multifaceted assessment of the quality of studies, focusing on NFB. The CRED-nf assesses the following domains: pre-experiment, control-groups, control measures, feedback specifications, outcome measures (brain and behavior), and data storage (Ros et al., 2020).

### Measures

The efficacy of NFB training in modulating brain circuits can be measured through changes in the EEG and in the reduction of behavioral symptoms (Rogala et al., 2016).

The EEG measurement is operationalized through a comparison of the amplitudes (absolute and relative) of the target EEG bands when the protocol is single-band or the amplitude ratios between bands in multi-band protocols (Rogala et al., 2016). The differences found in these parameters between the experimental and control groups are used as indicators of successful training. In contrast, changes in behavioral performance can be measured through instruments that assess the specific domain/symptom being trained (Rogala et al., 2016).

In this review, the articles analyzed included several effect measures and the following criteria were established to indicate NFB success:

1. Statistical significance (*p* < 0.05) was achieved through any statistical test, reflecting differences between groups or conditions (experimental vs. control) or pre- and post-treatment differences (in studies without a CG/condition). These effects were measured using either behavioral or neurophysiological assessments.
2. A significant change in all the groups, even if they did not differ from each other, suggesting that the EG achieved a significant result as well as the control group.

Studies that did not report significant results were qualified as ineffective. The results were tabulated to facilitate data organization.

### Certainty Assessment

The certainty assessment was done through the *Template for Developing Guidelines for the Evaluation of the Clinical Efficacy of Psychophysiological Interventions* (Vaque et al., 2002). These criteria aim to assess the extent to which an intervention can be considered effective and the level of trust that professionals and consumers (of NFB) can place in these judgments (Vaque et al., 2002). Therefore, each variable identified will be classified into one of five levels: level 1—Supported only by anecdotal reports and/or case studies in non-peer reviewed venues; 2—Possibly Efficacious; 3—Probably Efficacious; 4—Efficacious; and 5—Efficacious and Specific (Vaque et al., 2002).

## Results

### Study Selection

**Fig. 1:**
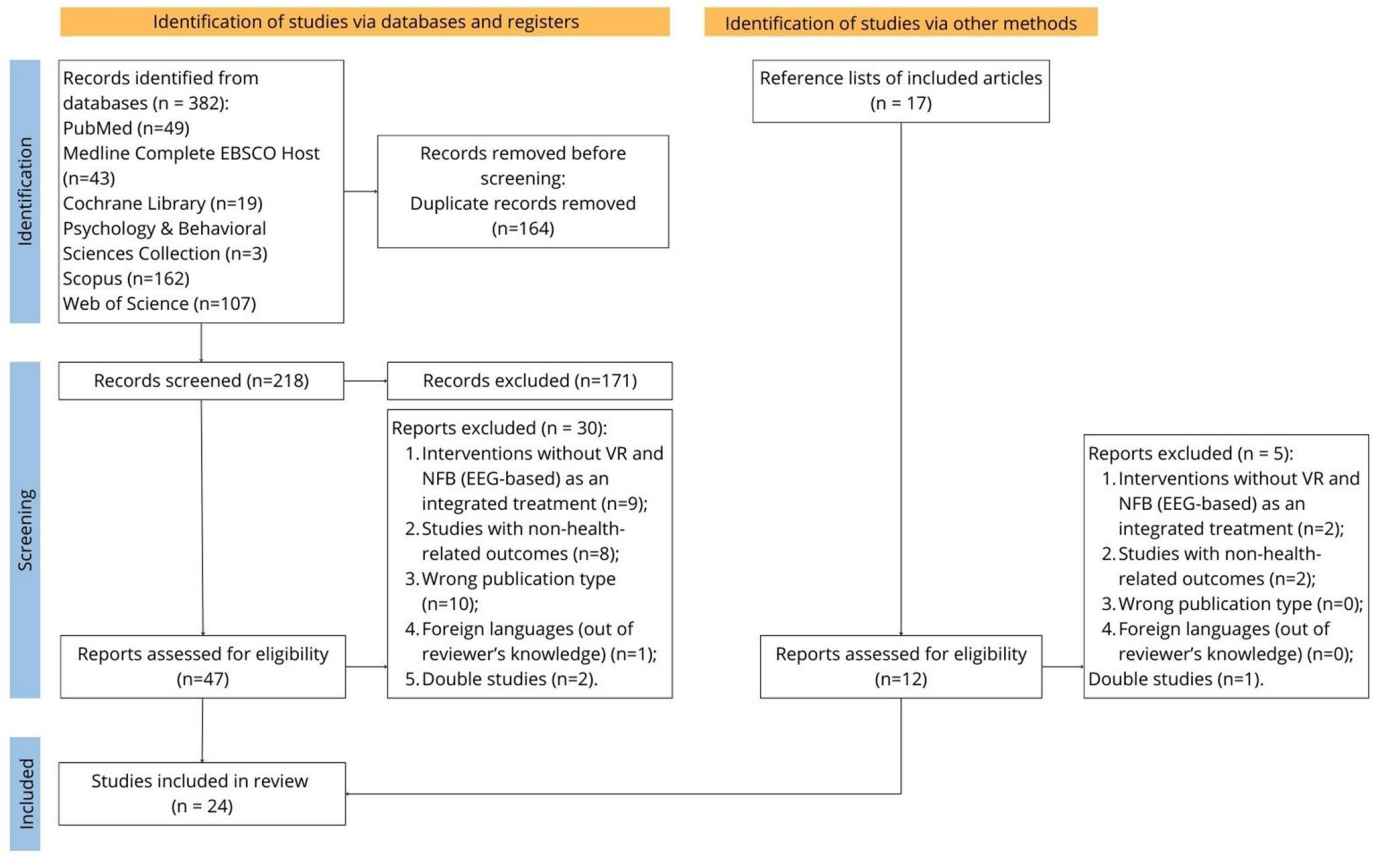
PRISMA flow diagram for study selection procedures

The flow diagram (Fig. 1) shows the study selection process. A total of 394 articles were identified by searching the databases (n = 382) and the reference lists of the articles included after the second screening (n = 12). Following the initial screening process, which involved the elimination of duplicates and articles that did not meet the eligibility criteria (based on a review of the title and abstract), 59 articles were selected for the second screening (full-text reading). These 59 articles were examined in detail, and 35 were excluded because they did not meet the inclusion criteria. Reasons to exclude studies from this review were: review studies, interventions without (immersive) VR and NFB (EEG-based) as an integrated treatment, studies with non-health-related outcomes, wrong publication type, foreign languages (not known to the reviewers)​, and double studies.

### Study Characteristics

The participants’ age ranged from 8 to 70 years, although three studies did not report this information. Eleven studies (45.8%) had samples with healthy participants and 13 (54.2%) had samples with some type of symptom/diagnosis. None of the studies compared the effects of treatment according to education level or age.

The sample sizes ranged from one to 100, given that this review included different types of study design. Three studies did not report or were unclear about the sample size.

The geographical distribution of the studies included China (four studies), Canada (three), England (two), the Republic of Korea (two), Finland (two), New York (two), Colombia (two), the United States of America (two), Austria (two), Chile (one), Italy (one), and Switzerland (one).

This review included six Randomized Controlled Studies (RCT), 14 non-randomized studies, one case study, and three mixed-methods studies. Given the topic in question and its innovative nature (hybrid approach), some of the studies were assumed to be feasibility/usability studies, pilot studies, proof-of-concept/principle, or exploratory studies.

Only three studies (Orakpo et al., 2021, 2022; Rolbiecki et al., 2023a, b) have reported the existence of conventional treatments concurrently with experimental treatments. No time period was set as an inclusion criterion to obtain a complete temporal evolution overview of this topic. Few studies have been published on this subject, with the highest number of publications in 2022. For further information see Supplementary Table 2.

### Risk of Bias in Studies

The risk of bias assessment for each study is summarized in Supplementary Table 3, where the results are presented according to the study design.

Highlighting the lack of relevant information to the risk of bias analysis of Randomized Controlled studies, three studies did not clearly report whether randomization was appropriately performed, and two did not clarify if there were complete outcome data and if outcome assessors were blinded to the intervention provided. Regarding non-randomized studies, the most frequently omitted information concerns confounding factors and the sample’s representativeness about the target population.

Of the 24 studies included, 8 had a quality assessment of 40%, 7 had 80%, 7 had 60% and two had 20%.

In the randomized studies, most unmet criteria were related to randomization and blinding, whereas in non-randomized studies, they were primarily related to the sample’s representativeness and the adequacy of the used measures.

Regarding the assessment conducted using the CRED-nf checklist (see Supplementary Table 4), the items that were least adequately addressed by the studies were as follows: *pre-register experimental protocol and planned analyses (1a); in clinical efficacy studies, employ a standard-of-care intervention group as a benchmark for improvement (2e); include measures of clinical or behavioral significance, defined *a priori*, and describe whether they were reached (6a)* (Ros et al., 2020)*.* On the other hand, the most fulfilled items were as follows: report the hardware and software used (4e); report the feedback modality and content (4c); and report and justify the reinforcement schedule (4b) (Ros et al., 2020). Only two studies (Berger & Davelaar, 2018; Berger et al., 2022) met more than 50% of the criteria.

### Results of Individual Studies

Given the heterogeneity of the study’s design, it was decided to provide a concise narrative description of the results found in the included articles and their practical implications. In addition, methodological approaches (e.g., NFB protocols, frequency and duration of sessions, and feasibility variables) that seemed relevant to the aim of the review were analyzed. Lastly, we addressed the synergy between the two techniques.

A brief analysis of the individual studies and the corresponding outcomes is presented in Table 1 and were analyzed for statistical significance.

**Table 1:** Key findings and practical implications

Authors and date	Comparator	Key findings	Practical implications
Benavides et al. (2022)	Pre- and post-comparison	Maslach Burnout Inventory: Burnout symptoms ↓	NFB integrated with VR-based relaxation therapy is a potential technique for brain’s self-regulation skills
Benedetti et al. (2014)	Pre- and post-comparison	Posner test: Acc ↓*; RT ↓*; CPT-II: Confidence Index ↓ (% of chance to present an attention disorder); Commissions ↓ (responding to the non-target stimulus or responding slowly); Variability ↓ (consistency of responding speed); Discrimination ↑ (score related to the ability to correctly identify target stimuli); D2: Commission ↑; Concentration ↑; Omission; Total correctly processed ↑; Total errors ↑; Total processed ↑	The combination of NFB with play therapy displays potential for increasing motivation, engagement and attention skills
Berger & Davelaar (2018)	3D VR environment (NFB + VR) versus 2D environment (NFB)	EEG (attentional control, frontal alpha oscillations and neural learning): between session learning—3D group ↑* vs. 2D group; within session learning—3D group ↑ and 2D group ↔; Stroop Task: Acc (affected by stoop effect conditions)—2D group ↓, 3D group ↔; RT ↓ (for both groups); Association* between an increase in frontal alpha activity and enhanced attentional processing	The NFB in a VR environment could be a promising tool for improving cognitive control and attentional performance
Chen et al. (2007)	3 states for each participant: Stable states; NFB + VR; NFB without VR	EEG: SMR power (attention enhancement)—NFB + VR condition ↑ versus NFB condition	A portable EEG biofeedback system can provide users with effective, user-friendly tools for creating flexible treatment at home; The VR offers to NFB an effective means of improving attention
Cho et al. (2004)	Control group (without intervention); VR group (HMD + Head tracker + NFB); Non-VR group (only computer monitor NFB)	CPT (attention): Number of Hits—↑* for both VR + NFB group and NFB group, NFB + VR group ↑ versus NFB group; RT—↓* for both groups; Perceptual sensitivity—↓* for both groups; Omission error—↓* for both groups, NFB + VR ↓*versus NFB and Control group; Commission error and response bias ↓* for both groups; EEG (regarding to attention): mean beta wave ratio in NFB training ↑*; VR + NFB group ↑ versus NFB group	It is important to understand the behavioral and mental characteristics of participants to determine the optimal number of sessions needed to validate long-term effects; Immersive VR can be applied to NFB for assessing and rehabilitating inattention and impulsiveness
Guedj et al. (2023)	Pre- and post-comparison	Sustained Attention Task: Acc and RT ↔; EEG (regarding to attention self-regulation): TBR ↓	The combination of EEG-NFB and VR offers a relatively short and playful therapeutic protocol aimed at teaching children with ADHD to regulate their own brain activity; This new EEG-NFB protocol combined with VR has the potential to improve the executive functions and behavioral skills of ADHD children
Gu & Frasson (2017)	Pre- and post-comparison	TItR—participants were divided into “Group never reached 0.6667” (meditation score) vs. “Group reached 0.6667” ↓; Meditation score– group reach 0.6667 ↑; HADS– A– “Group reached 0.6667” ↓; HADS– D– “Group reached 0.6667” ↓	The integration of NFB and VR technology is a promising approach with potential application in enhancing relaxation capacity
Järvelä et al. (2019)	Conditions: 4 solo (VR) scenarios (EEG + Respiration based adaptation; EEG; Respiration; none of the adaptations); and 4 dyadic (VR) scenarios, with an avatar (EEG + Respiration; EEG; Respiration; none of the adaptations)	Self-reported Empathy– Dyadic scenarios ↑* versus Solo scenarios; EEG + Respiration based adaptation ↑* vs. None of the adaptations; EEG + Respiration based adaptation ↑* versus Respiration	The use of physiological signals, such as breathing and brain activity, can provide real-time feedback and be useful in meditation contexts
Kosunen et al. (2016)	Conditions: Control Condition (meditation exercise on a computer screen without an HMD or NFB); HMD (without NFB); HMD + NFB	MEDEQ: Hindrances– Control ↑* vs HMD, HMD versus HMD + NFB ↔; Relaxation– Control ↓* versus HMD, HMD versus HMD + NFB ↔; Personal self– Control ↓* versus HMD, HMD versus. HMD + NFB ↔; Transpersonal qualities– Control ↓* versus HMD, HMD vs. HMD + NFB ↔; Transpersonal self– Control ↓* versus HMD, HMD versus HMD + NFB ↔	Integrating meditation with VR and NFB technology can enhance the meditation experience, promoting increased relaxation and a deeper sense of presence
Lu et al. (2022)	NFB session; Control session	EEG (Alpha band power reduction as a NFB index of the users’ attention): Alpha power at electrodes OZ, POZ and O1– EG ↓* versus CG; Attention level—EG ↑* vs. CG; Negative correlation* between Alpha power and attention performance	The results demonstrate the feasibility of NFB for VR attention training
Orakpo et al. (2021)	Pre- and post-comparison	Wong-Baker Pain Scale: Pain score ↓; ADL ↑; Pain-related anxiety ↓; Difficulty in sleep ↓; Pain-related fatigue and pain related depression ↓; PTSD flashbacks ↔	VF-NFB therapy demonstrates the potential to reduce opioid dependence and polypharmacy, and enhance resilience with sustained analgesia for a year; This treatment could be used as a complementary therapy, assisting psychiatrists and psychology practitioners to enhance the effectiveness of CBT for chronic pain
Orakpo et al. (2022)	Pre- and post-comparison	Wong-Baker Pain Scale: Pain ↓; Insomnia Severity Index: Insomnia ↓	VR-NFB at infra-low frequency is useful in reducing symptoms and eliminating dependence on sedatives
Rolbiecki et al. (2023a, 2023b)	Pre- and post-comparison	ESAS-r: Pain↓*; Tiredness↓*; Drowsiness↓; Nausea↓; Appetite↑; Shortness of breath↓; Depression↓; Anxiety↓; Well-being↓	This study provides a basis for a larger efficacy trial of an intervention that represents a non-pharmacological approach to managing cancer related symptoms
Tarrant et al. (2022)	EG—VR + NFB meditation experience; CG—standard guided audio-only meditation	Brunel Mood Scale: Tension↓*, Depression↓*, Happiness↑*, Calmness↑*, Anger↓*; Anger– CG ↑*versus EG, Vigor– EG↑* vs. CG	VR meditation combined with NFB can be an effective intervention to improve the mood of healthcare workers in a hospital setting. The immersive experience can enhance the benefits of meditation
Yan et al. (2008)	Pre- and post-comparison	IVA-CPT: Attention Quotient ↔ *; Visual Attention Quotient ↔ *; Auditory Attention Quotient ↔ *; Response Control Quotient ↔ *; Visual and Auditory Response Control Quotient ↔	The system has the potential to facilitate the treatment of ADHD in children The integration of electromyography could benefit in reducing hyperkinetic behavior
Yu et al. (2023)	Pre- and post-comparison	ERQ: Cognitive reappraisal↑*; Expressive suppression↓* DERS-16: Total score↓*; Nonacceptance ↔; Goals ↔; Impulse↑*; Strategies↑*; Clarity ↔; RSE: Self-esteem ↑*; HADS: Anxiety↓; Depression↓	VR combined with NFB can be an effective tool for promoting healthy emotional regulation skills
Zhang et al. (2019)	EG (participants with high-stress level, some of them with generalized anxiety disorder); CG (participants with low-level stress)	Game performance recordings: EG—Accuracy of multitasking↑, RT↓	An attention training system can be transformed into a VR game, augmented by an NFB BCI, to improve this cognitive ability
Abdessalem & Frasson (2017)	Pre- and post-comparison	EEG: Frustration ↔ *; Excitement ↔ *; Frustration↓* (when the game gets easy; Frustration↑* (when the game gets hard); Excitement↑* (when the game gets fast); Excitement↓* (when the game gets slow)	By using the NFB, this system can adjust the speed and difficulty of the VR game based on an assessment of the player’s emotional state (e.g. excitement and frustration), improving the user experience
Abdessalem et al. (2021)	3 immersive conditions: VR Train (VR only); Adaptive Music Therapy (VR + NFB); Intelligent Savannah Therapy (VR + NFB)	Mean performance of attention and memory exercises: VR Train– Frustration↓* (for all conditions); Memory↑* (for all conditions); Attention↑ (for Intelligent Savannah Therapy)	The use of an intelligent agent (i.e. EEG to monitor and evaluate emotions in real time) in VR environments could be promising in optimizing therapeutic effects in patients with subjective cognitive decline, especially in reducing negative emotions (frustration) and improving memory performance
Al-Shammari et al. (2022)	Pre- and post-comparison	EEG and performance analysis: Attention↑; Alpha ratio (increased alpha value leads to high attention performance)↑	A NFB system in a VR environment could be an effective approach to improving attention in adult ADHD patients
Berger et al. (2022)	3D condition (VR NFB); 2D condition (traditional NFB)	EEG: SMR power (state of relaxation and focus)– 3D↑* versus 2D; Theta power—3D↑* versus 2D; Beta power– 3D↑*, 2D ↔	The use of NFB in a VR (3D) environment can improve training performance compared to 2D feedback, suggesting that immersion can significantly influence the efficacy of NFB
Gruzelier et al. (2010)	Two training groups (ReaCTor group—VR + NFB; and NFB on screen group); CG (no training group)	The Acting Performance Scale: ReActor group—Imaginative Expression↑*, Imaginative Conviction↑*, Imaginative Characterization, Imagination↑*, Performance Overall↑, Well-Rounded Performance↑, Voice ↔, Movements ↔; Hamlet and Monologue scales: Training groups↑* versus CG (for Flow, Sense of control, Challenge-Skill Balance, Merging of Action, Awareness subscales); Reactor Group ↔ vs. NFB Group (for all subscales)	SMR NFB protocol training in immersive VR can significantly improve the artistic performance of actors. VR technology facilitates faster, and more effective learning compared to the traditional NFB approach. The ecological relevance of the training environment can increase the transfer of learned skills to real performance
Kober et al. (2016)	CG (2D NFB training); EG (3D VR + NFB group—patient A and B)	Visual Analogue Scale (VAS) and Questionnaire on Current Motivation: Motivation– EG↑* versus CG, Patient B↑*; Mood– Patient B↓*; Incompetence Fear—EG↑ versus CG; Mastery Confidence– EG versus CG↓; Interest—EG↑ versus CG; Challenge– EG ↔ versus CG; Feeling of Control– Patient A↑ versus CG, Patient B↓ versus CG; Concentration– EG ↔ vs CG	Implementing NFB scenarios in VR can improve the user experience and increase patient motivation and interest While VR can be a prevailing tool for engaging patients, it is crucial to consider users’ emotional reactions and unfamiliarity with this technology, especially in older samples or those less experienced with VR interfaces
Tarrant & Cope (2018)	Pre- and post-comparison	PANAS: Positive Affect– Participant 1 ↔, Participants 2,3 and 4↑; Negative Affect– Participant 1 ↔; Participants 2,3 and 4↓; STCI: State-Cheerfulness– Participants 1 and 3↓, Participant s 2 and 4↑; State-Bad Mood– Participant s 1,2 and 4↓, Participant 3 ↔	These results are promising as the intervention lasted only four to five minutes These results support the idea that consumer VR intervention can potentially have therapeutic effects and, as a relatively easy-to-use and inexpensive technology, can be used as a well-being tool at work and school, as a calming technique for people undergoing medical/dental procedures, or as a complement to traditional therapeutic interventions for anxiety or depression

↑ increase without statistical significance, ↓ decrease without statistical significance, ↔,no differences,↑* statistical significant increase, ↓* statistical significant decrease, ↔ * Statistical significant differences, *Acc* Accuracy, *RT* Reaction time, *SMR* Sensorimotor rhythm, *CPT* Continuous performance test, *TBR* Theta/Beta Ratio, *TItR* Time Interval to Relaxation, *HADS* Hospital Anxiety and Depression Scale A (Anxiety) and D (Depression), *HMD* Head mounted display, *MEDEQ* Meditation depth questionnaire, *CG* Control group, *EG* Experimental group, *ADL* Activities of daily living, *PTSD* Post-traumatic stress disease, *CBT* Cognitive behavioral therapy, *ESAS-r* Edmonton symptom assessment system revised version, *IVA*-*CPT* Integrated visual and auditory, continuous performance test, *ERQ* Emotiosn regulation questionnaire, *DERS* Difficulties in emotion regulation scale, *RSE* Rosenberg self-esteem scale, *BCI* Brain computer interaction, *PANAS* Positive and negative affect schedule, *STCI* State-trait-cheerfulness-inventory

**Fig. 2:**
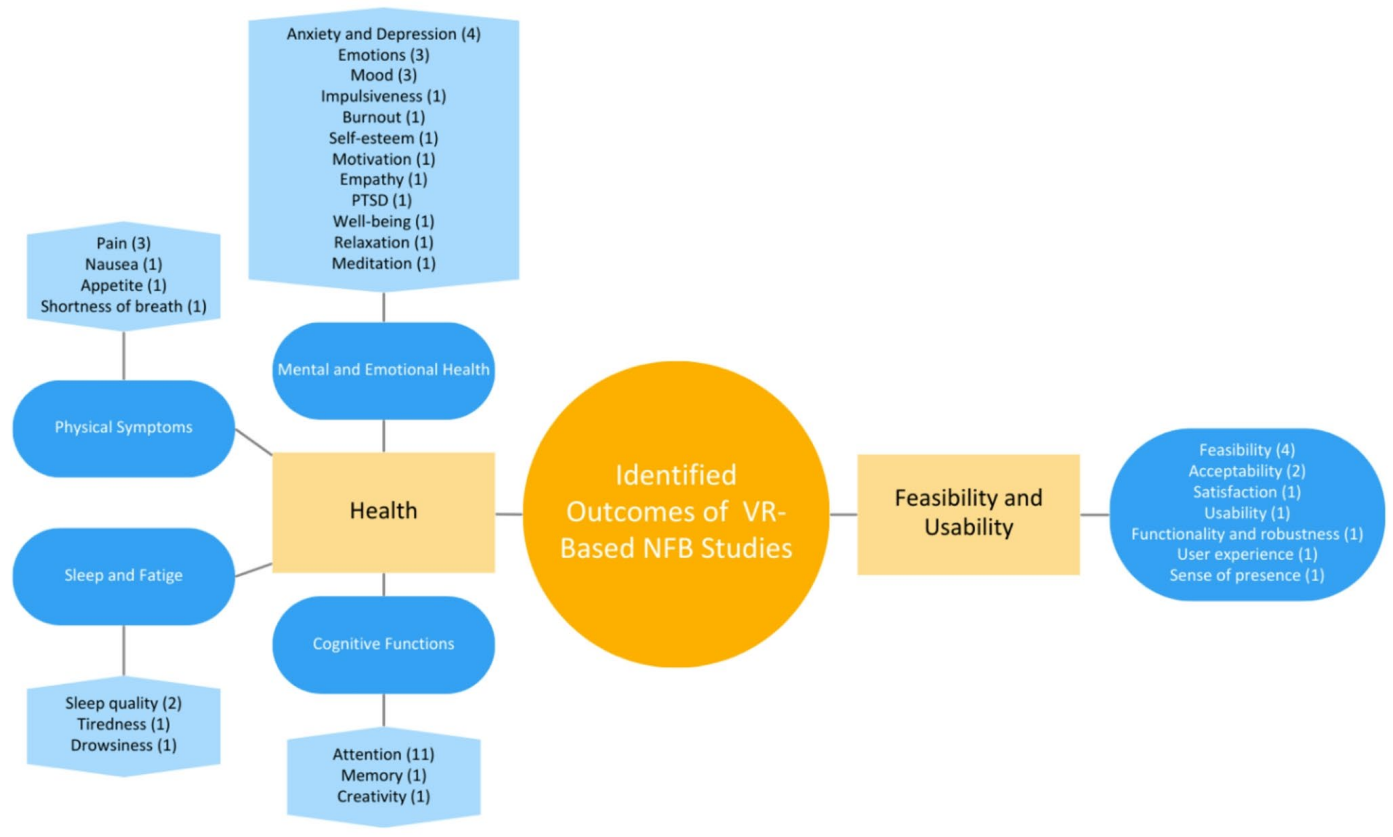
Variables identified and citation frequency in studies

After analyzing the results, 23 variables were identified: attention (in 11 studies); anxiety and depression (4); emotions (3); mood (3); pain (3); sleep quality (2); memory (1); impulsiveness (1); burnout (1); creativity (1); self-esteem (1); motivation (1); empathy (1); relaxation (1); meditation (1); post-traumatic stress disorder (PTSD) (1); tiredness (1); drowsiness (1); nausea (1); appetite (1); shortness of breath (1); and well-being (1). To facilitate ease of reading and understanding, all variables have been grouped into two main categories—health and feasibility/usability outcomes. Within the health category, subcategories were created to organize the large number of variables as shown in Fig. 2.

The outcomes were then categorized according to their level of efficacy and presented in their respective categories. The analyses were focused on the statistically significant outcomes.

#### Cognitive Outcomes

##### Attention

Eleven studies (45.8%) mentioned attention (alone or in combination with other variables) as an intervention domain of this treatment (Abdessalem et al., 2021; Al-shammari et al., 2022; Benedetti et al., 2014; Berger & Davelaar, 2018; Berger et al., 2022; Chen et al., 2007; Cho et al., 2004; Guedj et al., 2023; Lu et al., 2022; Yan et al., 2008; Zhang et al., 2019). Different terms have been used to refer to this outcome, including attention training (Benedetti et al., 2014), attentional control (Berger & Davelaar, 2018), SMR power regarding attention enhancement (Chen et al., 2007), SMR associated with the state of relaxation and focus (Berger et al., 2022), rehabilitation of inattention (Cho et al., 2004), theta/beta ratio (TBR) improvement regarding attention self-regulation (Guedj et al., 2023), alpha band power reduction as a NFB index of users’ attention (Lu et al., 2022), strengthening attention (Yan et al., 2008), attention training (Zhang et al., 2019), improving attention performance (Abdessalem et al., 2021), and attention improvement (Al-shammari et al., 2022).

Of the 11 studies, four were RCTs (Berger & Davelaar, 2018; Berger et al., 2022; Cho et al., 2004; Zhang et al., 2019) with CG/control condition, 6 were non-randomized studies (Abdessalem et al., 2021; Al-shammari et al., 2022; Chen et al., 2007; Guedj et al., 2023; Lu et al., 2022; Yan et al., 2008), three of which had a control condition, and one was a case study (Benedetti et al., 2014).

Three of the four RCTs (Berger & Davelaar, 2018; Berger et al., 2022; Cho et al., 2004) showed significant results. A study (Berger & Davelaar, 2018) with 22 participants indicated a notable enhancement (*p* = 0.04) in attentional levels in the 3D NFB (VR + NFB) group relative to the 2D group (conventional NFB), as evidenced by the Stroop task and alterations in the Gratton Effect (Berger & Davelaar, 2018).

Another study (Cho et al., 2004) found a significant effect in the VR + NFB group, compared to the NFB (without VR) and non-intervention groups, measured by the Continuous Performance Task (CPT), indicating a main effect significant (*p* < 0.01) for the VR + NFB group in number of hits, reaction time, perceptual sensitivity, and omission error scales. In addition to the behavioral results, neurophysiological results were also reported. A significant mean effect was found in beta wave ratio for group (*p* < 0.01) and time (*p* < 0.01). The VR group had higher rates than the non-VR group, except for one session. Additionally, the mean task completion time in NFB training showed significant statistical main effect of time (*p* < 0.05), and the completion time of the VR group decreased more noticeably than that of the non-VR group after the second session (Cho et al., 2004).

The third study (Berger et al., 2022) also yielded neurophysiological results, which revealed a significant main effect on training (*p* < 0.000) for the 3D feedback group, indicating a linear increase in SMR power. In contrast, the mixed effect model analysis for the 2D group revealed no significant effects (*p* = 0.106) (Berger et al., 2022), related to the effects of feedback modality (3D vs.2D) on Theta and Beta power. Post-tests revealed that the 3D group had significantly increased Beta power over the feedback runs (*p* < 0.001), while the 2D group did not (*p* = 0.102) (Berger et al., 2022). Of the 6 non-randomized studies, two (Lu et al., 2022; Yan et al., 2008) had significant results.

One of them (Lu et al., 2022) did an ANOVA analysis and showed that alpha power was lower in the EG than in the CG, as the protocol was to reduce the Alpha band power as an index of the users’ attention, and a significant main effect of group (*p* = 0.027) indicated that the attention level of the EG was higher than that of the CG.

The other study (Yan et al., 2008) showed a significant difference between pre- and post-treatment for attention quotient (*p* < 0.01), visual attention quotient (*p* < 0.01), and auditory attention quotient (*p* < 0.01), and response control quotient (*p* < 0.05). These differences were measured by the Integrated Visual and Auditory (IVA)—Continuous Performance Test (CPT).

With regard to the case study (Benedetti et al., 2014), the accuracy parameter, measured using Posner’s Spatial Cueing Task, showed a significant difference between the beginning and the end of the first phase of cognitive training (phase consisting of one training session and one rest period) (*p* < 0.001). A significant reduction in reaction time was observed as a result of the first experimental session (*p* < 0.001) and the second phase (*p* < 0.001).

In conclusion, these studies met the criteria for classifying this therapy as probably efficacious for attention (level 3).

##### Memory

For memory, a non-randomized study, with three conditions (VR—Train, VR + NFB—Adaptive Music Therapy, and Intelligent Savanah Therapy—VR + NFB with more intelligence component) performed paired t-tests on participants’ scores for three memory exercises, with pre and post comparisons (Abdessalem et al., 2021). The results were significant for the three conditions in one of the memory exercises (VR Train, *p* = 0.017; Adaptive Music Therapy, *p* = 0.0029; Intelligent Savanah Therapy, *p* = 0.0008). These findings indicate that NFB + VR is as effective as VR only. NFB + VR was considered possibly efficacious for memory improvement.

##### Creativity

Regarding creativity, an RCT (Gruzelier et al., 2010) was conducted with two EGs: ReaCTor group (VR + NFB) and NFB on-screen group (conventional NFB), and a CG (no training group) (Gruzelier et al., 2010). An ANOVA analysis showed a significant interaction between group and rating (*p* < 0.035). Post-hoc t-tests confirmed significant results (*p* < 0.05) for the ReaCTor group on the Imaginative Expression, Imaginative Conviction, Imaginative Characterization and Imagination subscales (*p* < 0.04). There were no statistical differences between the NFB groups. NFB  + VR treatment was classified as possibly efficacious for creativity.

#### Mental and Emotional Health Outcomes

##### Emotions

Three non-randomized controlled studies (12.5%) analyzed the variable emotions (Abdessalem & Frasson, 2017; Abdessalem et al., 2021; Yu et al., 2023) referring to the reduction of negative emotions (Abdessalem et al., 2021), emotional changes (Abdessalem & Frasson, 2017), and ability to down-regulate negative emotions (Yu et al., 2023).

One of the studies (Abdessalem et al., 2021) had three experimental conditions: VR Train (VR only), Adaptive Music Therapy (VR + NFB), and Intelligent Savanah Therapy (VR + NFB with the highest intelligence component). These three conditions significantly reduced participants’ frustration during and after the immersive experiences: VR train, *p* = 0.0011; Adaptive Music Therapy, *p* = 0.0011; and Intelligent Savanah Therapy, *p* = 0.0345. This result suggests that the intervention with NFB + VR was as effective as with VR alone.

Another study (Abdessalem & Frasson, 2017) described a neural agent as an intelligent system that adapts to the VR environment based on the user’s emotions. Significant results were observed in an ANOVA analysis showing that the EEG-measured levels of frustration and excitement were significantly different before and after the game intervention; excitement increased when the action was to accelerate the ambulance (*p* = 0.009) and decreased when the action was to slow down the ambulance (*p* = 0.005); frustration increased when the neural agent made the easer (*p* = 0.000) and decreased when it made the game easier (*p* = 0.002).

These results showed that when the neural agent adapts the game to the participant, it positively and significantly affects these emotions, adjusting the game speed and difficulty to the level of excitement and frustration (Abdessalem & Frasson, 2017).

The third study (Yu et al., 2023) found statistical significance in two emotion regulation strategies: an increase in cognitive reappraisal (*p* < 0.001) and a decrease in expressive suppression (*p* < 0.05). These outcomes were measured using the Emotion Regulation Questionnaire (ERQ). The Difficulties in Emotion Regulation Scale (DERS-16), which measures emotion regulation problems, showed a significantly lower result after treatment (*p* < 0.05).

This variable met the criteria for treatment classification as possibly efficacious (level 2).

##### Mood

Three studies (12.5%) analyzed the variable mood (Kober et al., 2016; Tarrant & Cope, 2018; Tarrant et al., 2022) and used terms including mood (Kober et al., 2016), positive changes in mood states (Tarrant & Cope, 2018), and improving mood states (Tarrant et al., 2022).

One of the non-randomized studies (Kober et al., 2016) had a sample of 9 patients with first-time stroke, two of which were the EG (3D NFB) and the others the CG (2D NFB). The only significant result was observed in one of EG patients, who showed a decrease after the first session (*p* < 0.10), measured using the Visual Analogue Scale (Kober et al., 2016). Another non-randomized study (Tarrant & Cope, 2018) did not achieve significant results.

The RCT (Tarrant et al., 2022) included an EG (VR + NFB meditation experience) and a CG (standard guided audio-only meditation). Using the Brunel Mood Ratings scale, significant group-by-time interactions were found for the happiness (*p* < 0.001), calmness (*p* < 0.001), vigor (*p *< 0.001), fatigue (*p* = 0.002), and confusion (*p* = 0.003) subscales; a significant main effect of time was found for the tension (*p* < 0. 001) and depression (*p* < 0.001) mood scales; and the main effect of time was also observed for happiness (*p* < 0.001) and calmness (*p* < 0.001), such that both groups showed an increase in scores on both scales from pre- to post-test. Additionally, a significant between-group effect for anger was noted (*p* < 0.001), with the CG rating higher on average than the EG at both time points. A significant main effect of time (*p* < 0.001) was also identified for the anger scale, indicating an overall decrease in both groups. There were also between-group effects for vigor, such that EG was rated higher than CG. In conclusion, EG was as effective as CG. This variable met the criteria for classifying this therapy as possibly efficacious.

##### Impulsiveness

Impulsiveness was analyzed in an RTC (Cho et al., 2004) with three groups: CG (no intervention), VR group (HMD plus Head Tracker and NFB), and non-VR group (computer monitor NFB). This variable was measured using the CPT, and statistical analysis found a main effect of time on the commission errors (*p* < 0.05) and response bias (*p* < 0.01) subscales (Cho et al., 2004). These results indicate that the treatment of NFB + VR is as effective as that of NFB alone and is classified as possibly efficacious for this variable.

##### Self-Esteem

Self-esteem was also analyzed in a non-randomized study showing significantly higher levels after NFB training (*p* < 0.05), accessed using the Rosenberg Self-Esteem Scale (RSE) (Yu et al., 2023). For self-esteem, the treatment was classified as possibly efficacious.

##### Motivation

The motivation variable was referenced in two studies (Guedj et al., 2023; Kober et al., 2016). Of these, one RCT (Kober et al., 2016) had 9 patients experiencing first-time stroke, divided into two groups: the EG (3D NFB), and the CG (2D NFB). A single case analysis revealed that motivation levels were higher and statistically significant for only one patient from EG during an intermediate session (*p* < 0.10). Another study (Guedj et al., 2023) did not find any statistically significant differences.

These findings suggest that this treatment was not empirically supported for motivation.

##### Empathy

The non-randomized study (Jarvela et al., 2019) which analyzed empathy, had 8 conditions: 4 solo VR scenarios (EEG plus respiration-based adaptation; EEG; respiration; and none of the adaptations); 4 dyadic VR scenarios, with an avatar (EEG plus respiration; EEG; respiration; and none of the adaptations). Empathy data were measured using self-reported data, respiration data records, and EEG physiological measures. Pairwise analyses revealed that there was a greater increase (*p* = 0.01) of self-reported empathy after sessions in which empathy was directed towards an avatar (represented by another human participant in a social or dyadic situation) than when it was directed towards an agent or a statue (a non-social or solo situation). Furthermore, the reported empathy levels were higher in both EEG adaptation conditions compared to the respiration adaptation only condition (*p* = 0.01). These findings provide evidence of this treatment as possibly efficacious for empathy.

##### Relaxation

The variable *relaxation* was analyzed in two studies (8.3%) (Berger et al., 2022; Gu & Frasson, 2017) with healthy adults, and only in one of them were the results statistically significant. The RCT (Berger et al., 2022) translated the variable relaxation to neurophysiological terms as SMR power, brain waves associated with a state of relaxation and focus. The study included a 3D condition (VR + NFB) and a 2D condition (conventional NFB). A linear mixed effects model for the dependent variable SMR power found a significant within-subject factor “runs” (that is, 3 min games) (*p* = 0.000) and a significant interaction effect between “feedback group” and “runs” (*p* = 0.001). Post-tests for each group revealed a significant main effect only for the 3D group (*p* < 0.000) (Berger et al., 2022). These studies met the criteria for classifying this therapy as possibly efficacious for relaxation.

##### Meditation

Concerning meditation, a non-randomized study (Kosunen et al., 2016) was conducted with control conditions (meditation on a computer screen without HMD or NFB), HMD condition (without NFB), and HMD plus NFB. The meditation experience was measured using the Meditation Depth Questionnaire (MEDEQ). It was divided into five phases of increasing depth: hindrances (negative feelings associated with the meditation experience), relaxation, personal self (self-perception effect caused by the practice of meditation), transpersonal qualities (essential qualities such as joy and love that can emerge from meditation), and transpersonal self (deeper levels of meditative experience). For all these factors, the differences between the HMD and the HMD plus NFB groups did not reach statistical significance (Kosunen et al., 2016). The same efficacy rating was given to NFB + VR for meditation (possibly efficacious).

#### Sleep and Fatigue Outcomes

The variable *tiredness* was analyzed in a clinical sample of 15 cancer patients (Rolbiecki et al., 2023a, 2023b) in a mixed methods study. The statistical analysis of the pre- and post-treatment comparisons demonstrated a statistically significant reduction in tiredness related to the experience of cancer and its treatment following 22 sessions (Rolbiecki et al., 2023a, 2023b). NFB treatment with VR for fatigue followed the same classification as the previous variables.

#### Physical Outcomes

Three studies (12.5%) analyzed the variable *pain* in clinical samples using pre/post comparisons and terms such as maintaining pain relief (Orakpo et al., 2021), improving central pain (Orakpo et al., 2022) and managing cancer-related pain (Rolbiecki et al., 2023a, 2023b).

Two were case reports (Orakpo et al., 2021, 2022) that did not perform statistical analyses to determine significance. However, they did find a trend towards pain reduction that was maintained for up to one year after a surgery procedure.

The third study (Rolbiecki et al., 2023a, 2023b), which used a mixed methods design and included 15 cancer patients, found a significant reduction in pain symptoms before and after treatment (*p* = 0.001), as measured by the Edmonton Symptom Assessment System revised version (ESAS-r) and the patient’s pain rate. Based on these results, this treatment was considered possibly efficacious for mood improvement.

#### Methodological Approaches

This section presents additional results which include feasibility and usability outcomes, outcome measures, comparators, NFB protocols, number of sessions, session frequency, and session duration (see Supplementary Table 5 for further details), as it is considered essential factors for innovative studies and contribute to the quality of efficacy studies.

##### Feasibility and Usability Variables

This category of variables addresses the viability of software, offering insights into the development of innovative interventions, including feasibility, sense of presence, acceptability, usability, user experience, satisfaction, functionality and robustness. Only 33,3% of the studies evaluated these factors (Chen et al., 2007; Guedj et al., 2023; Kober et al., 2016; Kosunen et al., 2016; Rolbiecki et al., 2023a, 2023b; Tarrant et al., 2022; Yan et al., 2008; Yu et al., 2023) (see Supplementary Table 5).

##### Number of Sessions, Duration and Frequency

Five studies (20.8%) did not report the number of sessions or were unclear on this data (Al-shammari et al., 2022; Jarvela et al., 2019; Kosunen et al., 2016; Tarrant & Cope, 2018; Zhang et al., 2019). The number of sessions varied from 1 to 20. Most studies applied the same number of sessions to each participant, except for two that did not set a fixed number (Gruzelier et al., 2010; Kober et al., 2016). The duration of the sessions exhibited significant variability, ranging from three to 60 min, and the frequency of sessions also presented notable variability, ranging from one to five times per week. It is noteworthy that most studies did not report this information.

#### Protocols

Eight studies (33.3%) did not report NFB protocols, and *attention* was the most explored variable concerning protocols. It was found that the used protocols differed from study to study whenever attention was the primary outcome, both in studies with healthy samples and in studies with samples with ADHD or other conditions. Therefore, different protocols were applied for the same variable, which did not affect the results. The use of psychological/cognitive tests or other non-neurophysiological measures was observed in some studies, while others included additional EEG data.

#### Neurofeedback and Virtual Reality Synergy

The innovative aspect of NFB treatment that is highlighted in this review is its association with VR, which provides 3D immersion as opposed to the conventional 2D NFB screen. A range of immersive scenarios was identified and categorized into three distinct groupings: diverse games [13 studies (Abdessalem & Frasson, 2017; Abdessalem et al., 2021; Al-shammari et al., 2022; Benedetti et al., 2014; Berger et al., 2022; Chen et al., 2007; Gruzelier et al., 2010; Kober et al., 2016; Orakpo et al., 2022; Tarrant et al., 2022; Yan et al., 2008; Yu et al., 2023; Zhang et al., 2019)]; cognitive tasks adapted to VR [4 studies (Berger & Davelaar, 2018; Cho et al., 2004; Guedj et al., 2023; Lu et al., 2022)]; and meditation/virtual relaxation [6 studies (Benavides et al., 2022; Gu & Frasson, 2017; Jarvela et al., 2019; Kosunen et al., 2016; Rolbiecki et al., 2023a, 2023b; Tarrant & Cope, 2018). One study (Orakpo et al., 2021) did not provide a description of the game or its VR scenario (see supplementary Table 6 for further information).

The games had different aims, including moving objects, racing cars, watching scenes/images to evoke emotions, and simulating the theater stage. The meditation and virtual relaxation-based studies incorporated a variety of scenarios, including Japanese gardens, waterfalls, the sea, beaches, and snow days, and some incorporated calming music and avatars. Therefore, the VR component used the visual and/or auditory senses. Two studies (Gruzelier et al., 2010; Guedj et al., 2023) used the Automatic Virtual Environment (CAVE) system in the immersive component.

Of the 24 studies included in this review, 12 had comparison groups in their sample composition. Of these 12 studies, only six (Berger & Davelaar, 2018; Berger et al., 2022; Chen et al., 2007; Cho et al., 2004; Gruzelier et al., 2010; Kosunen et al., 2016) had appropriate conditions/groups to compare, with adequate statistical analysis, whether NFB + VR was more effective than NFB alone. We can conclude that three of these studies (Berger & Davelaar, 2018; Berger et al., 2022; Chen et al., 2007) showed significant results in favor of this synergy of techniques, and the remaining three studies (Cho et al., 2004; Gruzelier et al., 2010; Kosunen et al., 2016), although showing positive results for both groups, the differences between them were not statistically significant.

## Discussion

This discussion compares the results of the present review with previous literature that focused exclusively on NFB or on VR, as there is a lack of reviews that have analyzed this integrated treatment.

The notion of clinical efficacy can be defined as the achievement of a desired therapeutic effect (Vaque et al., 2002). In this review, the efficacy of the 23 identified variables was measured in accordance with the guidelines for assessing the clinical efficacy of psychophysiological methods (Vaque et al., 2002). Subsequently, this treatment was classified as probably efficacious for attention, possibly efficacious for emotions, mood, pain, relaxation, impulsiveness, memory, self-esteem, creativity, empathy, meditation, and tiredness, and not empirically supported for anxiety, depression, relaxation, motivation, and burnout. This classification does not imply that the variables will not be assigned a higher efficacy classification in subsequent studies. The classification was developed with consideration for methodological rigor in the design and implementation and in the number of published studies. One of the conclusions drawn from this review is the need for more randomized controlled studies, as a standard reference for clinical validation, that bring these variables closer to higher levels of efficacy.

### Health Outcomes

Among the *cognitive variables* examined, attention emerged as the most prominent in terms of the number of studies published.

This treatment has been employed in the rehabilitation of patients with the aim of enhancing their attention performance. Attention was the variable that achieved the highest level of efficacy, and it was considered probably efficacious (level 3 of 5). No conclusions can be drawn comparing this intervention to conventional therapies due to inadequate trial controls. The significant results found in 6 (54.5%) of the 11 studies that analyzed attention are inconsistent, confirming previous literature. For example, a meta-analysis of meta-analyses concluded that NFB has consistently demonstrated its efficacy in the treatment of ADHD (which includes attention) over a decade (Riesco-Matías et al., 2019). On the other hand, another meta-analysis of the effect of digital interventions on ADHD found that NFB did not significantly improve symptoms of inattention (Liu et al., 2024).

For VR, significant improvements in attention were found in children with ADHD compared to CGs in a systematic review (Corrigan et al., 2023). Of the 11 studies that analyzed attention, three did not report the NFB protocols, and the remaining used different protocols. The standard protocols for ADHD are Theta/Beta, SMR, and NFB of slow cortical potentials (Arns et al., 2014), however, four studies did not follow these protocols, which did not lead to negative results. With NFB, a personalized approach can improve clinical outcomes (Arns et al., 2014). For example, some cases of attention deficit disorder are associated with hyperactivity, while others are not, and this can be a crucial factor in determining the most appropriate protocol (Arns et al., 2014).

The only study related to memory applied this treatment to people with SCD, and the results were significant for both VR and VR + NFB. The literature is broader (e.g., post-stroke participants) (Maggio et al., 2019; Yi et al., 2023), with significant results (Laborda-Sánchez & Cansino, 2021; Trambaiolli et al., 2021; Vilou et al., 2023), and even more selective in terms of the memory type (Gruzelier, 2014; Yeh et al., 2021, 2022). For VR, however, previous studies on working and episodic memory have not found significant results (Gómez-Cáceres et al., 2022).

Regarding the category of *mental and emotional outcomes*, although emotions variable was only analyzed in three non-randomized studies, the results were statistically significant. This finding aligns with a previous review that found VR to be beneficial for emotion regulation, as it provides the opportunity to learn complex emotion regulation strategies in digital environments (Montana et al., 2020).

The significant results for mood also confirm previous evidence of NFB benefits (Domenicucci et al., 2022) and VR with a large effect size in stroke patients (Gao et al., 2021).

Although impulsiveness was mentioned in only one study, it has been extensively studied over the years, as part of ADHD symptoms, in NFB field. Previous reviews have reported efficacy with medium (Van Doren et al., 2019) and large (Arns et al., 2009; Barlas, 2021) effect sizes. For VR, data are also promising, but lack scientific robustness (Gardini et al., 2022).

Only one non-randomized study showed significance for motivation. This outcome can be considered a health-related variable, as it can have a major influence, for example, in the learning context (Jiang & Fryer, 2023) and its potentiating effect on treatment (Guedj et al., 2023; Kober et al., 2016). Motivational factors have been identified as moderate predictors of NFB success (Kadosh & Staunton, 2019).

The study on empathy found significant results consistent with a previous study on VR and increased empathy. VR allows participants to immerse themselves in the perspective of others (Lee et al., 2024).

Only one RCT (Berger et al., 2022) found statistical significance for relaxation. This is consistent with previous studies that, while recognizing its benefits, warn of the need for further research (Riches et al., 2021). Relaxation can be used to relieve symptoms (with VR) (Riches et al., 2021) and to improve performance in athletes (with NFB) (Mikicin et al., 2015), but these data are still scarce.

Two meanings have been suggested in studies on meditation: one aimed at facilitating the meditative process (Navarrete et al., 2021; Navarro-Haro et al., 2017; Seabrook et al., 2020), and the other aimed at reducing symptoms (Dreesmann et al., 2023; Hargett et al., 2022; Kim et al., 2024; Venuturupalli et al., 2019). NFB has also been associated with meditation, as external feedback makes it perceptible and improves the performance of this experience (Nieto-Vallejo et al., 2021; Pandey et al., 2023; Van Lutterveld et al., 2017), although the study in this review did not identify any significant differences between the meditation groups (VR vs. VR + NFB).

For the group of variables in the *sleep and fatigue category*, the tiredness has been explored in the field of NFB for symptom management and relief in cancer patients (Luctkar-Flude & Groll, 2015), with promising results as found in this review, although with limited scientific robustness.

Lastly, in the category of *physical variables*, significant results were only observed in one (Rolbiecki et al., 2023a, 2023b) of the three studies with regard to pain, since the other two were case reports. Previous reviews have shown positive effects of NFB on pain (Diotaiuti et al., 2024; Hetkamp et al., 2019; Patel et al., 2020), highlighting the potential for pain relief in people with chronic pain. Current evidence is still limited for robust recommendations on the most effective protocols (Roy et al., 2020). Furthermore, the possible analgesic effects of NFB on pain are short term, according to a narrative review (Schuurman et al., 2024). In previous VR studies it has also found significant results with benefits for pain in acute, chronic and procedural pain conditions, particularly in patients reporting moderate to severe pain (Lier et al., 2023), and in perioperative and periprocedural contexts for chronic pain (Viderman et al., 2023). However, the limited capacity to increase pain tolerance and the action mechanisms were based on distraction and activation of multiple senses. The activation of the visual cortex and the integration of other senses allows VR to change the manipulation of nociceptive stimuli (Viderman et al., 2023).

### Feasibility and Usability Outcomes

The feasibility of studies is often assessed through pilot studies, which are small-scale tests of methods and procedures designed to test the acceptability of approaches before they are applied on a larger scale. This provides relevant data to inform possible improvements and increase the success (Teresi et al., 2022). Guidelines have been developed (Teresi et al., 2022) that address methods for testing the feasibility of implementing interventions. Some of these were followed in the studies of this review, such as: (1) choice of conceptual and psychometric measures that are appropriate, acceptable and relevant to the target population; (2) adherence and involvement of participants in the intervention; and (3) acceptability. Data from this variable could provide valuable insights and improvements for future studies, bringing research closer to more robust clinical results. Due to its pertinence to the innovative treatment under discussion, we concluded that a small number of studies (33.3%) analyzed feasibility/usability related factors, and this was a negative finding for investigation.

#### Neurofeedback and Virtual Reality Synergy

Three (Berger & Davelaar, 2018; Berger et al., 2022; Chen et al., 2007) of the six studies with adequate comparative analysis showed significant results in favor of this synergy of techniques, and the remaining three studies (Cho et al., 2004; Gruzelier et al., 2010; Kosunen et al., 2016), although showing positive results for both groups, there were no statistically significant differences. All three studies that yielded statistical significance of this technique’s synergy assessed attention. In contrast, one of the studies showing non-statistically significant results also included the attention variable, suggesting a lack of consistency.

It is imperative to acknowledge that, despite the absence of control groups, 12 studies employed pre- and post-comparisons and obtained favorable results for this synergy, including five with statistical significance (Abdessalem & Frasson, 2017; Benedetti et al., 2014; Rolbiecki et al., 2023a, 2023b; Yan et al., 2008; Yu et al., 2023) and the rest without statistical significance or adequate statistical analyses.

These promising findings can be attributed to the fact that VR has been demonstrated that immersion increases motivation, interest, compliance, and more realistic and explanatory feedback of what happens in the brain during training (rather than abstract and non-intuitive feedback). Previous studies have shown that adding VR to biofeedback interventions can lead to increased motivation, engagement, and user experience (Lüddecke & Felnhofer, 2022). Additionally, the experience of presence leads users to respond to sensory data as if it were real (Kober et al., 2016; Marzbani et al., 2016; Rezaie Khosravi & Mahmoodi, 2018; Yan et al., 2008).

Of the studies that used different game scenarios, no correlation was found between what was presented in the game and the symptoms/targets of treatment. A relevant question for NFB studies is whether users can transfer the mental state they achieve in training to other contexts without real-time feedback on their brain activation. In the specific case of children with ADHD, it is relevant to be able to simulate a virtual classroom in order to facilitate training and the transfer of results to real-life school (Kober et al., 2024), as identified in one of the studies in this review which had a CAVE system (Guedj et al., 2023). This leads us to question whether the variety of immersive scenarios could influence the desired outcomes, as they range from different games to virtual meditation.

The potential of VR extends beyond the scope of the NFB by enabling the customization of feedback, contributing to an overall positive experience (Kober et al., 2024).

In short, statistical significance was observed in half of the studies that met the necessary comparison conditions, and the other half showed positive results, although without statistical differences. This review demonstrated the promising potential of these two techniques’ synergy in alleviating health-related symptoms compared to the use of NFB alone. The main obstacle to some protocols reaching higher levels of evidence is the large number of uncontrolled studies and the lack of precise replications due to the heterogeneity of protocol details, duration, improvement measures, control conditions and sample characteristics (Vaque et al., 2002).

### Limitations of the Evidence Included in the Review

Not restricting the inclusion criteria to RCTs allowed a broad view of published studies on the topic, but the range of designs made it impractical to conduct meta-analyses and generalize the results. Only 25% of the studies were RCTs, the gold standard for evaluating the efficacy of interventions, which was a limitation in assessing the level of evidence for the intervention studied (Vaque et al., 2002), as this is a relatively new topic and some studies were exploratory.

Evidence for the therapeutic efficacy of EEG NFB is limited by methodological weaknesses, corroborated through CRED-nf checklist, which can lead to inconsistent results and justify the skepticism of researchers and clinicians regarding this treatment (Micoulaud-Franchi et al., 2015). The risk of bias assessment showed that only 7 studies (30%) met 80% of the quality criteria, which was the highest score; and for the quality of NFB protocols, 57% was the highest score, found in two studies (8%). Despite the use of these two measures to obtain a complementary and more multifaceted assessment, they were employed in an uncorrected fashion, lacking adequate control for integrating both results.

### Implications and Future Work

Although few studies have been conducted, most are promising, with possible clinical and research implications. This review suggests a range of therapeutic applications, from relieving symptoms to improving performance in healthy people, at any age.

VR has the potential to allow immersion in controlled virtual environments, anywhere (Bell et al., 2020), which may improve the access to psychological therapies (Freeman et al., 2017). Additionally, NFB + VR has the potential to reduce opioid dependence, polypharmacy, and to be used as a non-invasive complementary therapy, assisting psychologists and psychiatrists to enhance the effectiveness of Cognitive Behavioral Therapy (Orakpo et al., 2021, 2022) or other health professionals in different medical conditions (e.g., cancer) (Rolbiecki et al., 2023a, 2023b).

However, researchers should consider the ecological validity, cost, access, scientific evidence, technical challenges, and ethical implications of VR (Bell et al., 2020).

Future studies could customize the VR scenarios to allow a connection between them and the user’s symptoms and facilitate the transfer of NFB results. Moreover, future studies should use more robust methods to investigate the underlying mechanisms of NFB, including appropriate comparator groups, and determine whether these combination techniques are more effective than NFB alone and whether they enhance the efficacy of first-line therapies. It is imperative to consider feasibility and usability metrics considering the pioneering nature of this hybrid treatment.

International guidelines for standardization of trial design and reporting, such as the CRED-nf checklist, are highly recommended to be followed.

## Conclusion

The present review suggests that NFB EEG-based combined with immersive VR is probably efficacious for enhancing attention and possibly efficacious for emotions, mood, pain, relaxation, impulsiveness, memory, self-esteem, creativity, empathy, meditation, and tiredness.

These results are promising and lead to exploring the benefits of the synergy between these two techniques in a wide range of health areas, including physical, mental, cognitive and emotional problems, and optimizing cognitive performance in healthy people.

The findings of this study indicate that NFB EEG-based with VR is a promising approach. Nevertheless, additional research employing robust and rigorous methodologies is needed to more thoroughly understand the mechanisms underlying the value of adding VR to the NFB. Such knowledge would facilitate the replication of existing studies and the validation of clinical findings.

## Electronic Supplementary Material

Below is the link to the electronic supplementary material.

Supplementary Material 1

## Data Availability

No datasets were generated or analysed during the current study.
