# Supplementary material for: The Efficacy of Virtual Reality-Based EEG Neurofeedback in Health-Related Symptoms Relief: A Systematic Review
Source: Appl Psychophysiol Biofeedback. 2025 Jul 26;50(4):613–33. doi: 10.1007/s10484-025-09730-0 (PMC12686002; doi:10.1007/s10484-025-09730-0)
Supplement: Supplementary file 1 — Supplementary Material 1 [file 10484_2025_9730_MOESM1_ESM.docx]

**Supplementary table 1.** Search Terms

|  | | **Free terms** | **MeSH** | **PBSC** | |
| --- | --- | --- | --- | --- | --- |
| Concept 1 | | “virtual reality” OR “VR” OR “virtual environment” OR “immersive environment” OR “immersive virtual reality” | “Virtual Reality” | “Virtual Reality” | |
| Concept 2 | | neurofeedback OR “NFB” OR “NF” OR “neurofeedback training” OR “NFT” OR “EEG-based neurofeedback” OR “EEG Feedback” OR “Electroencephalography Biofeedback” OR Neurotherapy | Neurofeedback | ---------------------------- | |
| Concept 3 | | health OR “health symptoms” OR “health related symptoms” OR anxiety OR Anxiousness OR depression OR “depressive symptoms” OR “emotion* regulation” OR “Emotion* Self-Regulation” OR memory OR “verbal fluency” OR multitasking OR attention OR inattention OR impulsive* OR pain OR “motor capabilities” OR “Motor Skills” OR “cognitive symptoms” OR “emotional symptoms” OR “behavioral symptoms” OR “behavioural symptoms” OR “physical symptoms” OR “Neurobehavioral Manifestations” OR “Internalizing Symptoms” OR “Emotional Responses” OR “Motor ability” | Anxiety OR “anxiety disorders” OR Depression OR “emotional regulation” OR Memory OR “Memory Disorders” OR “Pain management” OR attention OR pain OR “Pain Perception” OR “Motor Skills” OR “Neurobehavioral Manifestations” OR “Behavioral Symptoms” | “Mental depression”  OR Anxiety OR “anxiety disorders” OR “emotion regulation” OR Memory OR “Memory Disorders” OR attention OR “Attention-deficit hyperactivity disorder” OR Pain OR “Pain management” OR “Pain Perception” OR “Motor ability” | |
| PubMed | | | | | |
| Research | Terms | | | | Results |
| #1 | **("virtual reality"[Title/Abstract] OR "VR"[Title/Abstract] OR "virtual environment"[Title/Abstract] OR "immersive environment"[Title/Abstract] OR "immersive virtual reality"[Title/Abstract]) OR ("Virtual Reality"[MeSH Terms])** | | | | 27 974 |
| #2 | **(neurofeedback[Title/Abstract] OR "NFB"[Title/Abstract] OR "NF"[Title/Abstract] OR "neurofeedback training"[Title/Abstract] OR "NFT"[Title/Abstract] OR "EEG-based neurofeedback"[Title/Abstract] OR "EEG Feedback"[Title/Abstract] OR "Electroencephalography Biofeedback"[Title/Abstract] OR Neurotherapy[Title/Abstract]) OR (Neurofeedback[MeSH Terms])** | | | | 146 934 |
| #3 | **(((((((((((((health[Title/Abstract] OR "health symptoms"[Title/Abstract] OR "health related symptoms"[Title/Abstract] OR anxiety[Title/Abstract] OR Anxiousness[Title/Abstract] OR depression[Title/Abstract] OR "depressive symptoms"[Title/Abstract] OR "emotion* regulation"[Title/Abstract] OR "Emotion* Self-Regulation"[Title/Abstract] OR memory[Title/Abstract] OR "verbal fluency"[Title/Abstract] OR multitasking[Title/Abstract] OR attention[Title/Abstract] OR inattention[Title/Abstract] OR impulsive*[Title/Abstract] OR pain[Title/Abstract] OR "motor capabilities"[Title/Abstract] OR "Motor Skills"[Title/Abstract] OR "cognitive symptoms"[Title/Abstract] OR "emotional symptoms"[Title/Abstract] OR "behavioral symptoms"[Title/Abstract] OR "behavioural symptoms"[Title/Abstract] OR "physical symptoms"[Title/Abstract] OR "Neurobehavioral Manifestations"[Title/Abstract] OR "Internalizing Symptoms"[Title/Abstract] OR "Emotional Responses"[Title/Abstract] OR "Motor ability"[Title/Abstract]) OR (Anxiety[MeSH Terms])) OR ("anxiety disorders"[MeSH Terms])) OR (Depression[MeSH Terms])) OR ("emotional regulation"[MeSH Terms])) OR (Memory[MeSH Terms])) OR ("Memory Disorders"[MeSH Terms])) OR ("Pain management"[MeSH Terms])) OR (attention[MeSH Terms])) OR (pain[MeSH Terms])) OR ("Pain Perception"[MeSH Terms])) OR ("Motor Skills"[MeSH Terms])) OR ("Neurobehavioral Manifestations"[MeSH Terms])) OR ("Behavioral Symptoms"[MeSH Terms])** | | | | 5 276 689 |
| #4 | **((("virtual reality"[Title/Abstract] OR "VR"[Title/Abstract] OR "virtual environment"[Title/Abstract] OR "immersive environment"[Title/Abstract] OR "immersive virtual reality"[Title/Abstract]) OR ("Virtual Reality"[MeSH Terms])) AND ((neurofeedback[Title/Abstract] OR "NFB"[Title/Abstract] OR "NF"[Title/Abstract] OR "neurofeedback training"[Title/Abstract] OR "NFT"[Title/Abstract] OR "EEG-based neurofeedback"[Title/Abstract] OR "EEG Feedback"[Title/Abstract] OR "Electroencephalography Biofeedback"[Title/Abstract] OR Neurotherapy[Title/Abstract]) OR (Neurofeedback[MeSH Terms]))) AND ((((((((((((((health[Title/Abstract] OR "health symptoms"[Title/Abstract] OR "health related symptoms"[Title/Abstract] OR anxiety[Title/Abstract] OR Anxiousness[Title/Abstract] OR depression[Title/Abstract] OR "depressive symptoms"[Title/Abstract] OR "emotion* regulation"[Title/Abstract] OR "Emotion* Self-Regulation"[Title/Abstract] OR memory[Title/Abstract] OR "verbal fluency"[Title/Abstract] OR multitasking[Title/Abstract] OR attention[Title/Abstract] OR inattention[Title/Abstract] OR impulsive*[Title/Abstract] OR pain[Title/Abstract] OR "motor capabilities"[Title/Abstract] OR "Motor Skills"OR "cognitive symptoms"[Title/Abstract] OR "emotional symptoms"[Title/Abstract] OR "behavioral symptoms"[Title/Abstract] OR "behavioural symptoms"[Title/Abstract] OR "physical symptoms"[Title/Abstract] OR "Neurobehavioral Manifestations"[Title/Abstract] OR "Internalizing Symptoms"[Title/Abstract] OR "Emotional Responses"[Title/Abstract] OR "Motor ability"[Title/Abstract]) OR (Anxiety[MeSH Terms])) OR ("anxiety disorders"[MeSH Terms])) OR (Depression[MeSH Terms])) OR ("emotional regulation"[MeSH Terms])) OR (Memory[MeSH Terms])) OR ("Memory Disorders"[MeSH Terms])) OR ("Pain management"[MeSH Terms])) OR (attention[MeSH Terms])) OR (pain[MeSH Terms])) OR ("Pain Perception"[MeSH Terms])) OR ("Motor Skills"[MeSH Terms])) OR ("Neurobehavioral Manifestations"[MeSH Terms])) OR ("Behavioral Symptoms"[MeSH Terms]))** | | | | 49 |
| Medline Complete EBSCO Host | | | | | |
| Research | Terms | | | | Results |
| S1 | TI ( “virtual reality” OR VR OR “virtual environment” OR “immersive environment” OR “immersive virtual reality” ) OR AB ( “virtual reality” OR “VR” OR “virtual environment” OR “immersive environment” OR “immersive virtual reality” ) OR MH “Virtual Reality” | | | | 25 864 |
| S2 | TI ( neurofeedback OR "NFB" OR "NF" OR “neurofeedback training” OR "NFT" OR “EEG-based neurofeedback” OR “EEG Feedback” OR “Electroencephalography Biofeedback” OR Neurotherapy ) OR AB ( neurofeedback OR "NFB" OR "NF" OR “neurofeedback training” OR "NFT" OR “EEG-based neurofeedback” OR “EEG Feedback” OR “Electroencephalography Biofeedback” OR Neurotherapy ) OR MH Neurofeedback | | | | 134 630 |
| S3 | TI ( health OR “health symptoms” OR “health related symptoms” OR anxiety OR Anxiousness OR depression OR “depressive symptoms” OR “emotion* regulation” OR “Emotion* Self-Regulation” OR memory OR “verbal fluency” OR multitasking OR attention OR inattention OR impulsive* OR pain OR “motor capabilities” OR “Motor Skills” OR “cognitive symptoms” OR “emotional symptoms” OR “behavioral symptoms” OR “behavioural symptoms” OR “physical symptoms” OR “Neurobehavioral Manifestations” OR “Internalizing Symptoms” OR “Emotional Responses” OR “Motor ability” ) OR AB ( health OR “health symptoms” OR “health related symptoms” OR anxiety OR Anxiousness OR depression OR “depressive symptoms” OR “emotion* regulation” OR “Emotion* Self-Regulation” OR memory OR “verbal fluency” OR multitasking OR attention OR inattention OR impulsive* OR pain OR “motor capabilities” OR “Motor Skills” OR “cognitive symptoms” OR “emotional symptoms” OR “behavioral symptoms” OR “behavioural symptoms” OR “physical symptoms” OR “Neurobehavioral Manifestations” OR “Internalizing Symptoms” OR “Emotional Responses” OR “Motor ability” ) OR MH ( Anxiety OR “anxiety disorders” OR Depression OR “emotional regulation” OR Memory OR “Memory Disorders” OR “Pain management” OR attention OR pain OR “Pain Perception” OR “Motor Skills” OR “Neurobehavioral Manifestations” OR “Behavioral Symptoms” ) | | | | 4 612 113 |
| S4 | S1 AND S2 AND S3 | | | | 43 |
| Cochrane Library | | | | | |
| **Research** | **Terms** | | | | **Results** |
| #1 | (“virtual reality” OR “VR” OR “virtual environment” OR “immersive environment” OR “immersive virtual reality”):ti,ab,kw | | | | 7 659 |
| #2 | MeSH descriptor: [Virtual Reality] explode all trees | | | | 1 042 |
| #3 | #1 OR #2 | | | | 7 662 |
| #4 | (neurofeedback OR “NFB” OR “NF” OR “neurofeedback training” OR “NFT” OR “EEG-based neurofeedback” OR “EEG Feedback” OR “Electroencephalography Biofeedback” OR Neurotherapy):ti,ab,kw | | | | 3 497 |
| #5 | MeSH descriptor: [Neurofeedback] explode all trees | | | | 446 |
| #6 | #4 OR #5 | | | | 3 497 |
| #7 | (health OR “health symptoms” OR “health related symptoms” OR anxiety OR Anxiousness OR depression OR “depressive symptoms” OR emotion* NEXT regulation OR Emotion* Self-Regulation OR memory OR “verbal fluency” OR multitasking OR attention OR inattention OR impulsive* OR pain OR “motor capabilities” OR “Motor Skills” OR “cognitive symptoms” OR “emotional symptoms” OR “behavioral symptoms” OR “behavioural symptoms” OR “physical symptoms” OR “Neurobehavioral Manifestations” OR “Internalizing Symptoms” OR “Emotional Responses” OR “Motor ability”):ti,ab,kw | | | | 642 302 |
| #8 | MeSH descriptor: [Anxiety] explode all trees | | | | 13 405 |
| #9 | MeSH descriptor: [Anxiety Disorders] explode all trees | | | | 9 311 |
| #10 | MeSH descriptor: [Depression] explode all trees | | | | 18 859 |
| #11 | MeSH descriptor: [Emotional Regulation] explode all trees | | | | 234 |
| #12 | MeSH descriptor: [Memory] explode all trees | | | | 9 921 |
| #13 | MeSH descriptor: [Memory Disorders] explode all trees | | | | 1 522 |
| #14 | MeSH descriptor: [Pain Management] explode all trees | | | | 5 153 |
| #15 | MeSH descriptor: [Attention] explode all trees | | | | 6 761 |
| #16 | MeSH descriptor: [Pain] explode all trees | | | | 75 424 |
| #17 | MeSH descriptor: [Pain Perception] explode all trees | | | | 938 |
| #18 | MeSH descriptor: [Motor Skills] explode all trees | | | | 2 289 |
| #19 | MeSH descriptor: [Neurobehavioral Manifestations] explode all trees | | | | 11 653 |
| #20 | MeSH descriptor: [Behavioral Symptoms] explode all trees | | | | 32 308 |
| #21 | #7 OR #8 OR #9 OR #10 OR #11 OR #12 OR #13 OR #14 OR #15 OR #16 OR #17 OR #18 OR #19 OR #20 | | | | 663126 |
| #22 | #3 AND #6 AND #21 | | | | 19 |
| Psychology & Behavioral Sciences Collection | | | | | |
| **Research** | **Terms** | | | | **Results** |
| S1 | TI ( “virtual reality” OR “VR” OR “virtual environment” OR “immersive environment” OR “immersive virtual reality” ) OR AB ( “virtual reality” OR “VR” OR “virtual environment” OR “immersive environment” OR “immersive virtual reality” ) OR SU “Virtual Reality” | | | | 2 968 |
| S2 | TI ( neurofeedback OR “NFB” OR “NF” OR “neurofeedback training” OR “NFT” OR “EEG-based neurofeedback” OR “EEG Feedback” OR “Electroencephalography Biofeedback” OR Neurotherapy ) OR AB ( neurofeedback OR “NFB” OR “NF” OR “neurofeedback training” OR “NFT” OR “EEG-based neurofeedback” OR “EEG Feedback” OR “Electroencephalography Biofeedback” OR Neurotherapy ) | | | | 1 187 |
| S3 | TI ( health OR “health symptoms” OR “health related symptoms” OR anxiety OR Anxiousness OR depression OR “depressive symptoms” OR “emotion* regulation” OR “Emotion* Self-Regulation” OR memory OR “verbal fluency” OR multitasking OR attention OR inattention OR impulsive* OR pain OR “motor capabilities” OR “Motor Skills” OR “cognitive symptoms” OR “emotional symptoms” OR “behavioral symptoms” OR “behavioural symptoms” OR “physical symptoms” OR “Neurobehavioral Manifestations” OR “Internalizing Symptoms” OR “Emotional Responses” OR “Motor ability” ) OR AB ( health OR “health symptoms” OR “health related symptoms” OR anxiety OR Anxiousness OR depression OR “depressive symptoms” OR “emotion* regulation” OR “Emotion* Self-Regulation” OR memory OR “verbal fluency” OR multitasking OR attention OR inattention OR impulsive* OR pain OR “motor capabilities” OR “Motor Skills” OR “cognitive symptoms” OR “emotional symptoms” OR “behavioral symptoms” OR “behavioural symptoms” OR “physical symptoms” OR “Neurobehavioral Manifestations” OR “Internalizing Symptoms” OR “Emotional Responses” OR “Motor ability” ) OR SU ( “Mental depression” OR Anxiety OR “anxiety disorders” OR “emotion regulation” OR Memory OR “Memory Disorders” OR attention OR “Attention-deficit hyperactivity disorder” OR Pain OR “Pain management” OR “Pain Perception” OR “Motor ability” ) | | | | 348 118 |
| S4 | S1 AND S2 AND S3 | | | | 2 |
| Scopus | | | | | |
| **Research** | **Terms** | | | | **Results** |
| 1 | ( TITLE-ABS-KEY ( "virtual reality" OR "VR" OR "virtual environment" OR "immersive environment" OR "immersive virtual reality" ) AND TITLE-ABS-KEY ( neurofeedback OR "NFB" OR "NF" OR "neurofeedback training" OR "NFT" OR "EEG-based neurofeedback" OR "EEG Feedback" OR "Electroencephalography Biofeedback" OR neurotherapy ) AND TITLE-ABS-KEY ( health OR "health symptoms" OR "health related symptoms" OR anxiety OR anxiousness OR depression OR "depressive symptoms" OR "emotion* regulation" OR "Emotion* Self-Regulation" OR memory OR "verbal fluency" OR multitasking OR attention OR inattention OR impulsive* OR pain OR "motor capabilities" OR "Motor Skills" OR "cognitive symptoms" OR "emotional symptoms" OR "behavioral symptoms" OR "behavioural symptoms" OR "physical symptoms" OR "Neurobehavioral Manifestations" OR "Internalizing Symptoms" OR "Emotional Responses" OR "Motor ability" ) ) | | | | 162 |
| Web of Science | | | | | |
| **Research** | **Terms** | | | | **Results** |
| 1 | TS=(“virtual reality” OR “VR” OR “virtual environment” OR “immersive environment” OR “immersive virtual reality”) AND TS=(neurofeedback OR “NFB” OR “NF” OR “neurofeedback training” OR “NFT” OR “EEG-based neurofeedback” OR “EEG Feedback” OR “Electroencephalography Biofeedback” OR Neurotherapy) AND TS=(health OR “health symptoms” OR “health related symptoms” OR anxiety OR Anxiousness OR depression OR “depressive symptoms” OR “emotion* regulation” OR “Emotion* Self-Regulation” OR memory OR “verbal fluency” OR multitasking OR attention OR inattention OR impulsive* OR pain OR “motor capabilities” OR “Motor Skills” OR “cognitive symptoms” OR “emotional symptoms” OR “behavioral symptoms” OR “behavioural symptoms” OR “physical symptoms” OR “Neurobehavioral Manifestations” OR “Internalizing Symptoms” OR “Emotional Responses” OR “Motor ability”) | | | | 107 |

**Supplementary table 2.** Study characteristics

| **Authors and date** | **Study design** | **Sample size** | **Participants' symptoms and/or inclusion/exclusion criteria** | **Age range** |
| --- | --- | --- | --- | --- |
| Benavides et al., 2022 | Quantitative Non-Randomized | 4 | - Healthcare workers in pandemic context for SarsCov 2; - Burnout. | 38, 58, 32, 38 |
| Benedetti et al., 2014 | Qualitative | 1 | - Frontal syndrome (medium–high severity) with frontal lobes damage and impairments related to attention and concentration, to the ability to support a cognitive activity over time and switch from one line of thought to another, loss of spontaneous initiative (apathy), depressive mood, irritability, aggression, and lack of awareness of his own cognitive disorders and sexual disinhibition. | 36 |
| Berger and Davelaar, 2018 | Quantitative Randomized | 22 | - Healthy; - Exclusion criteria: participants prone to motion or VR sickness (identified with an online screening questionnaire); susceptibility to migraines; diagnosis of ADHD or epilepsy; priorpsychiatric treatment; current pharmacological treatment (especially benzodiazepine-based); and high levels of anxiety and stress. | Mean age = 35.2  SD = 8.8 |
| Chen et al., 2007 | Quantitative Non-Randomized | 3 | - Healthy | Not reported |
| Cho et al., 2004 | Quantitative Randomized | 28 | - Social problems (had committed crimes and isolated in a reformatory); some difficulty in learning, inattentive, impulsive, hyperactive and distracted (not officially diagnosed as having ADHD). | From 14 to 18 |
| Guedj et al., 2023 | Quantitative Non-Randomized | 4 | - Healthy; - Inclusion/exclusion criteria: typically developing, without ADHD diagnosis. | Mean age = 9.46  SD = 1.23 |
| Gu and Frasson, 2017 | Quantitative Non-Randomized | 6 | - Healthy - Inclusion/exclusion criteria: do not have attention deficit disorder, dyslexia, dyspraxia and so on; have normal hearing; never suffered a cranial trauma; not taking any medications acting on the nervous system; able to understand English; willing to stay on and complete the 8-session, training twice a week for one month. | Mean age = 29.67 SD = 4.84 |
| Järvelä et al., 2019 | Quantitative Non-Randomized | 42 | - Healthy - Inclusion criteria: over 18 years old; right-handed; fluent in Finnish; without any diagnosed neurological or neuropsychological disorders. | Mean age = 27.09 SD = 6.61 |
| Kosunen et al., 2016 | Quantitative Non-Randomized | 43 | - Healthy university students | Mean age = 28.7 (from 20 to 48) |
| Lu et al., 2022 | Quantitative Non-Randomized | 18 | - Healthy | Mean age = 23.67 (from 20 to 28) |
| Orakpo et al., 2021 | Mixed Methods | 1 | - Chronic pain secondary to spondylolisthesis with cervical, thoracic, and lumbar disc herniations after a motor vehicle accident with comorbid depression, anxiety, sleep deprivation, difficulty with activities of daily living (ADL), and inability to participate in physical therapy. | 55 |
| Orakpo et al., 2022 | Mixed methods | 1 | - Moderate-severe insomnia secondary to chronic lower back pain and sciatica | 31 |
| Rolbiecki et al., 2023 | Mixed methods | 15 | - Cancer-related pain patients; - Inclusion criteria: currently experiencing cancer-related pain; at least 18 years old; receiving anti- cancer treatment (chemotherapy) at the infusion clinic; willing to participate in the VR experience and complete follow-up surveys and interviews; cognitively intact; and without auditory or visual deficit. | Mean age = 52.4 SD = 11.8 (from 36 to 70) |
| Tarrant et al., 2022 | Quantitative Randomized | 100 | - Healthy frontline healthcare workers in a hospital; - Inclusion criteria: at least 18 years old; - Exclusion criteria: any history of seizures; current blindness; or severe eye impairment which would limit the ability to engage in the VR experience; or history of motion sickness. | Experimental group: Mean age = 42.16 SD = 14.4  Control group: Mean age = 40.9 SD = 13.9 |
| Yan et al., 2008 | Quantitative Non-Randomized | 7 | - ADHD diagnosed clinically children | From 8 to 12 |
| Yu et al., 2023 | Quantitative Non-Randomized | 8 | - Healthy college students | Mean age = 24.3 SD = 3.5 |
| Zhang et al., 2019 | Quantitative Randomized | Not clear | - Experimental group: participants with high-stress level [some with Generalized Anxiety Disorder (GAD)]; - Control group: paticipants with low-level stress | Not reported |
| Abdessalem and Frasson, 2017 | Quantitative Non-Randomized | 20 | - Healthy | Mean age = 31.05 SD = 4.9619 |
| Abdessalem et al., 2021 | Quantitative Non-Randomized | 57 | - Subjective Cognitive Decline (SCD); - Inclusion criteria: older than aged 60 of age; francophone; normal or correct-to-normal vision; normal hearing; met the Consortium for the Early Identification of Alzheimer’s Disease-Quebec (CIMA-Q) criteria for SCD. | Virtual Reality Train:  Mean age = 69.68  SD = 5.49;  Adaptive Music Therapy:  Mean age = 72.26  SD = 5.82;  Intelligent Savannah Therapy:  Mean age = 71  SD = 8.39. |
| Al-shammari et al., 2022 | Quantitative Non-Randomized | Not reported | - ADHD adults patients | Not reported |
| Berger et al ., 2022 | Quantitative Randomized | 61 | - Young healthy adults; - Inclusion criteria: absence of neurological or psychiatric diseases and age between 18 and 34 years old. | Mean age = 23.48 SD = 3.49 |
| Gruzelier et al., 2010 | Quantitative Randomized | 15 | - Healthy class of second year drama students | Mean age = 20 |
| Kober et al., 2016 | Quantitative Non-Randomized | 9 | - Patients with first-time stroke; - Exclusion criteria: drug treatment that interferes with the vigilance state; visual hemi-neglect; dementia (MMSE<24); psychiatric disorders such as depression or anxiety; concomitant neurological disorders (e.g. Parkinson disease or visual-reflex epilepsy, aphasia); insufficient motivation and cooperation. - Inclusion criteria: had normal or corrected-to-normal vision and hearing. | From 37 to 76 |
| Tarrant and Cope, 2018 | Quantitative Non-Randomized | 4 | - Firefighters - Mental health diagnoses: participant 1 - Post Traumatic Stress Desease (PTSD); 2 - Depression; 3 - None; 4 - Anxiety, Depression and PTSD). | Mean age = 39.5 SD = 5.6 |

**Supplementary table 3.** Risk of Bias Assessment using Mixed Methods Appraisal Tool

| **Category of study designs** | **Methodological quality criteria** | **Responses** | | | |
| --- | --- | --- | --- | --- | --- |
| Sceeening questions  (for all types) | S1. Are there clear research questions? | Yes | No | Can’t tell | Comments |
|  | S2. Do the collected data allow to address the research questions? |  |  |  |  |
| 1. Qualitative | 1.1. Is the qualitative approach appropriate to answer the research question? |  |  |  |  |
|  | 1.2. Are the qualitative data collection methods adequate to address the research question? |  |  |  |  |
|  | 1.3. Are the findings adequately derived from the data? |  |  |  |  |
|  | 1.4. Is the interpretation of results sufficiently substantiated by data? |  |  |  |  |
|  | 1.5. Is there coherence between qualitative data sources, collection, analysis and interpretation? |  |  |  |  |
| 2. Quantitative  randomized controlled  trials | 2.1. Is randomization appropriately performed? |  |  |  |  |
|  | 2.2. Are the groups comparable at baseline? |  |  |  |  |
|  | 2.3. Are there complete outcome data? |  |  |  |  |
|  | 2.4. Are outcome assessors blinded to the intervention provided? |  |  |  |  |
|  | 2.5 Did the participants adhere to the assigned intervention? |  |  |  |  |
| 3. Quantitative non-  randomized | 3.1. Are the participants representative of the target population? |  |  |  |  |
|  | 3.2. Are measurements appropriate regarding both the outcome and intervention (or exposure)? |  |  |  |  |
|  | 3.3. Are there complete outcome data? |  |  |  |  |
|  | 3.4. Are the confounders accounted for in the design and analysis? |  |  |  |  |
|  | 3.5. During the study period, is the intervention administered (or exposure occurred) as intended? |  |  |  |  |
| 5. Mixed methods | 5.1. Is there an adequate rationale for using a mixed methods design to address the research question? |  |  |  |  |
|  | 5.2. Are the different components of the study effectively integrated to answer the research question? |  |  |  |  |
|  | 5.3. Are the outputs of the integration of qualitative and quantitative components adequately interpreted? |  |  |  |  |
|  | 5.4. Are divergences and inconsistencies between quantitative and qualitative results adequately addressed? |  |  |  |  |
|  | 5.5. Do the different components of the study adhere to the quality criteria of each tradition of the methods involved? |  |  |  |  |

| Methodological Quality Criteria | | | | | | | | | |
| --- | --- | --- | --- | --- | --- | --- | --- | --- | --- |
| **Qualitative studies** | | | | | | | | | |
| **Study (author/s and date)** | S1 | S2 | 1.1. | 1.2. | 1.3. | 1.4. | 1.5. | Results  (% of quality criteria met) | Comments |
| Benedetti et al., 2014 | ✔ | ✔ | ✔ | ? | ? | ✔ | ? | 40% | Does not clearly identify qualitative data collection methods |
| **Quantitative Randomized Studies** | | | | | | | | | |
| **Study (author/s and date)** | S1. | S2. | 2.1. | 2.2. | 2.3. | 2.4. | 2.5 | Results  (% of quality criteria met) | Comments |
| Berger and Davelaar, 2018 | ✔ | ✔ | **?** | ✔ | ✔ | ✘ | ✘ | 80% | The participant retention rate was around 81% |
| Cho et al., 2004. | ✔ | ✔ | ? | ✔ | ✔ | ? | ✔ | 60% |  |
| Tarrant et al., 2022 | ✔ | ✔ | ✘ | ✔ | ✔ | ✘ | ✔ | 60% | Assignment that is predictable such as using odd and even numbers is not appropriate |
| Zhang et al., 2019 | ✔ | ✔ | ✘ | ? | ? | ? | ✔ | 20% |  |
| Berger et al., 2022 | ✔ | ✔ | ? | ✔ | ✔ | ✔ | ✔ | 80% |  |
| Gruzelier et al., 2010 | ✔ | ✔ | ? | ✔ | ? | ✔ | ? | 40% | Training groups underwent between 7 and 10 half-hour sessions of training. |
| **Quantitative Non-Randomized Studies** | | | | | | | | | |
| **Study (author/s and date)** | S1. | S2. | 3.1. | 3.2. | 3.3. | 3.4. | 3.5. | Results  (% of quality criteria met) | Comments |
| Benavides et al., 2022 | ✔ | ✔ | ✘ | ✔ | ✘ | **?** | ✔ | 40% | The cases were selected based on the number of sessions completed and not on the level of stress presented by the patients. |
| Chen et al., 2007 | ✔ | ✔ | ✘ | ✘ | ✔ | **?** | ✔ | 40% | The study does not use cognitive measures, only EEG |
| Guedj et al., 2023 | ✔ | ✔ | ✘ | ✔ | ✘ | **?** | ✔ | 40% |  |
| Gu and Frasson, 2017 | ✔ | ✔ | ✘ | ✘ | ✔ | **?** | ✔ | 40% |  |
| Järvelä et al., 2019 | ✔ | ✔ | ✘ | ✘ | ✔ | ✔ | ✔ | 60% |  |
| Kosunen et al., 2016 | ✔ | ✔ | **?** | ✔ | ✔ | ✔ | ✔ | 80% |  |
| Lu et al., 2022 | ✔ | ✔ | **?** | ✘ | ✔ | ✘ | ✔ | 40% | The study does not use cognitive measures, only EEG |
| Yan et al., 2008 | ✔ | ✔ | ✘ | ✔ | ✔ | ✔ | ✔ | 80% |  |
| Yu et al., 2023 | ✔ | ✔ | ✘ | ✔ | ✔ | **?** | ✔ | 60% |  |
| Abdessalem and Frasson, 2017 | ✔ | ✔ | **?** | ✘ | ✔ | ✘ | ✔ | 40% | The study does not use psychological measures, only EEG |
| Abdessalem et al., 2021 | ✔ | ✔ | ✘ | ✔ | ✔ | **?** | ✔ | 60% | The study sample had subjective cognitive decline, which suggests that it may not be representative of all Alzheimer's patients, but rather of a subgroup |
| Al-shammari et al., 2022 | ✔ | ✔ | **?** | ✘ | **?** | **?** | ✔ | 20% | The sample size was not reported in the study |
| Kober et al., 2016 | ✔ | ✔ | ✘ | ✔ | ✔ | ✔ | ✔ | 80% |  |
| Tarrant and Cope, 2018 | ✔ | ✔ | ✘ | ✔ | ✔ | ✔ | ✔ | 80% |  |
| **Mixed Methods Studies** | | | | | | | | | |
| **Study (author/s and date)** | S1. | S2. | 5.1. | 5.2. | 5.3. | 5.4. | 5.5. | Results  (% of quality criteria met) | Comments |
| Rolbiecki et al., 2023 | ✔ | ✔ | ✔ | ✔ | ✔ | ✔ | ✘ | 80% |  |
| Orakpo et al., 2021 | ✔ | ✔ | ✘ | ✔ | ✔ | ✔ | ✘ | 60% |  |
| Orakpo et al., 2022 | ✔ | ✔ | ✘ | ✔ | ✔ | ✔ | ✘ | 60% |  |

Legend: ✔ - Yes; ✘ - No; ? – Can’t tell

**Supplementary table 4**. Assessment of neurofeedback protocol quality with CRED–nf checklist

|  | | | **Reported on page #** | | | | | | | | | | | | | | | | | | | | | | | |
| --- | --- | --- | --- | --- | --- | --- | --- | --- | --- | --- | --- | --- | --- | --- | --- | --- | --- | --- | --- | --- | --- | --- | --- | --- | --- | --- |
| **Domain** | Item # | Checklist item | Benavides et al., 2022 | Benedetti et al., 2014 | Berger and Davelaar, 2018 | Chen et al., 2007 | Cho et al., 2004 | Guedj et al., 2023 | Gu and Frasson, 2017 | Järvelä et al., 2019 | Kosunen et al., 2016 | Lu et al., 2022 | Orakpo et al., 2021 | Orakpo et al., 2022 | Rolbiecki et al., 2023 | Tarrant et al., 2022 | Yan et al., 2008 | Yu et al., 2023 | Zhang et al., 2019 | Abdessalem and Frasson, 2017 | Abdessalem et al., 2021 | Al-shammari et al., 2022 | Berger et al., 2022 | Gruzelier et al., 2010 | Kober et al., 2016 | Tarrant and Cope, 2018 |
| **Pre-experiment** | 1a | Pre-register experimental protocol and planned analyses | N | N | N | N | N | N | N | N | N | N | N | N | N | N | N | N | N | N | N | N | N | N | N | N |
|  | 1b | Justify sample size | N | N/A | N | N/A | N | N | N | N | N | N | N/A | N/A | N | 4 | N | N | N | N | N | N | N | N | N | N |
| **Control groups** | 2a | Employ control group(s) or control condition(s) | N/A | N/A | 190 | 172 | 520 | N | N | 4217 | 213 | 5141 | N/A | N/A | N | 4 | N | N | 161 | N | 80 | N | 2-3 | 113 | 85 | N |
|  | 2b | When leveraging experimental designs where a double-blind is possible, use a double-blind | N/A | N/A | N | N | N | N/A | N/A | N | N | 5141 | N/A | N/A | N/A | N | N/A | N/A | N | N/A | N | N/A | 3 | N | N | N/A |
|  | 2c | Blind those who rate the outcomes, and when possible, the statisticians involved | N/A | N/A | N | N | N | N/A | N/A | N | N | N | N/A | N/A | N/A | N | N/A | N/A | N | N/A | N | N/A | N | 113 | N | N/A |
|  | 2d | Examine to what extent participants and experimenters remain blinded | N/A | N/A | N | N | N | N/A | N/A | N | N | N | N/A | N/A | N/A | N | N/A | N/A | N | N/A | N | N/A | 3 | 113 | N | N/A |
|  | 2e | In clinical efficacy studies, employ a standard-of-care intervention group as a benchmark for improvement | N/A | N/A | N | N | N | N/A | N/A | N | N | N | N/A | N/A | N/A | N | N/A | N/A | N | N/A | N | N/A | N | N | N | N/A |
| **Control measures** | 3a | Collect data on psychosocial factors | N | N | N | N | N | 9 | N | N | N | N | N | N | N | N | N | N | N | N | N | N | N | N | 88 | N |
|  | 3b | Report whether participants were provided with a strategy | N | 240 | 195 | N | N | N | 4-5 | 4216 | N | N | N | N | N | N | N | 1-2 | N | N | N | N | N | N | N | N |
|  | 3c | Report the strategies participants used | N | N | 195 | N | N | 9 | N | N | N | N | N | N | N | N | N | 3 | N | N | N | N | N | N | N | N |
|  | 3d | Report methods used for online-data processing and artifact correction | N | N | 191 | N | N | 6-7 | N | N | N | 5141 | N | N | N | N | 168 | 2 | 160 | N | N | 19103 | 3-4 | N | 87 | 61 |
|  | 3e | Report condition and group effects for artifacts | N | N | N | N | N | N | N | N | N | N | N | N | N | N | N | N | N | N | N | N | 4 | N | N | N |
| **Feedback specifications** | 4a | Report how the online-feature extraction was defined | N | N | 191 | 168-169 | 520-521 | 11 | N | 4217 | 212 | 5141 | N | N | N | 4 | 169 | 2-3 | 160 | N | N | 19108 | 3 | 113 | 86 | 60 |
|  | 4b | Report and justify the reinforcement schedule | N | 239 | 192 | 169 | 520-521 | 3 | N | N | N | 5141 | N | N | 293 | 4 | 169 | 3 | 161 | 137-138 | 79 | N | 3-4 | 113 | 87 | 58 |
|  | 4c | Report the feedback modality and content | N | 239 | 192 | 168 | 521 | 8 | 4 | 4215 | N | 5141 | N | N | 293 | 4 | 169 | 3 | 161 | 137-138 | 79 | 19107 | 3-4 | 113 | 87 | 58 |
|  | 4d | Collect and report all brain activity variable(s) and/or contrasts used for feedback, as displayed to experimental participants | N | N | 191 | N | 521-522 | 8 | N | N | N | N | N | N | N | N | N | N | 161 | N | N | N | 3, 6-7 | 113 | N | N |
|  | 4e | Report the hardware and software used | N | 239 | 192-194 | 170-171 | 520 | 6 | 5-6 | 4214 | 210, 213 | 5141 | 2 | N | 292-293 | 4-5 | N | 2 | 160 | 139 | 82-83 | 19102-19103 | 3 | 113 | 86 | 59-60 |
| **Outcomes measures: Brain** | 5a | Report neurofeedback regulation success based on the feedback signal | N | 241  -243 | 193 | 172 | 522-524 | 14-15 | N | 4218 | N | N | N | N | N | N | 171 | N | N | N | N | 19107-19108 | 4-9 | 114 | 89 | 61-63 |
|  | 5b | Plot within-session and between-session regulation blocks of feedback variable(s), as well as pre-to-post resting baselines or contrasts | N | N | 192-194 | N | 522-524 | 14-15 | N | N | N | N | N | N | N | N | N | N | 161 | 140-141 | N | 19107-19108 | N | N | 90 | N |
|  | 5c | Statistically compare the experimental condition/group to the control condition(s)/group(s) (not only each group to baseline measures) | N/A | N/A | 193-194 | N | 522-524 | N/A | N/A | 4218 | 213-215 | 5142-5143 | N/A | N | N/A | 6-8 | N/A | N/A | 161 | N/A | 90-91 | 19104-19109 | 4-7 | 114-115 | 89 | N/A |
| **Outcomes measures: Behavior** | 6a | Include measures of clinical or behavioral significance, defined a priori, and describe whether they were reached | N | N | N | N | N | N | N | N | N | N | N | N | N | N | N | N | N | N | N | N | N | N | N | N |
|  | 6b | Run correlational analyses between regulation success and behavioral outcomes | N | N | 194 | N | N | N | N | N | N | 5142-5143 | N | N | N | N | N | N | N | N | N | N | N | 115 | N | 63 |
| **Data storage** | 7a | Upload all materials, analysis scripts, code, and raw data used for analyses, as well as final values, to an open access data repository, when feasible | N | N | N | N | N | N | N | N | N | N | N | N | N | 10 | N | N | N | N | N | N | 9 | N | N | N |
| Yes % | | | 0%  *^1^ | 22%  *^1^ | 57% | 26%* | 39% | 43%* | 13% | 30% | 17% | 39% | 4%  *^1^ | 0%  *^1^ | 13%  * | 30%  * | 22%  * | 30% | 39% | 17% | 22% | 30%  * | 57% | 48%  * | 43%* | 30%* |

**Legend**: N = No; N/A = Not applicable; * = Feasibility/prof-of-concept/prof-of-principle/exploratory/pilot study (small sample size); *1 = Case study or case report

**Supplementary table 5.** Outcomes and protocol information

| **Author(s) and date** | **Outcome(s)** | **Outcome measures** | **Comparator** | **Neurofeedback protocol information** |
| --- | --- | --- | --- | --- |
| Benavides et al., 2022 | - Prevention and management of Burnout Syndrome | - Maslach Burnout Inventory | Pre-post comparison | Alpha training Deep relaxation |
| Benedetti et al., 2014 | - Attention training | - Posner's Spatial Cueing Task; - D2 Test; - Continuous Performance Test – II. | Pre-post comparison | Not reported |
| Berger and Davelaar, 2018 | - Attentional control  (frontal alpha oscillations); - Neural learning | - EEG; - Stroop task; - Gratton effect. | 3D virtual reality environment vs. 2D environment | Increase level of alpha amplitude at prefrontal cortex |
| Chen et al., 2007 | - SMR power (Attention enhancement); - Functionality and robustness of EEG device | - EEG (SMR power) | 3 conditions: Stable states; Attention with VR; Attention without VR | SMR |
| Cho et al., 2004 | - Rehabilitation of inattention and impulsiveness | -Continuous performance task (CPT): Attention (number of hits, reaction time, perceptual sensitivity, omission error); Impulsivity (commission error and response bias). | CG (without intervention); VR group (HMD + Head tracker); Non-VR group (only computer monitor with a fixed viewpoint) | Reinforce of beta wave ratio |
| Guedj et al., 2023 | - Acceptation, satisfaction, and sustaining motivation; - Approach feasibility; - Theta/Beta Ratio (TBR) improvement (regarding to attention self-regulation) | - Sustained Attention Task; - Neurofeedback Transfer Task; - Satisfaction survey; - EEG and fMRI recordings. | Pre-post comparison | TBR |
| Gu and Frasson, 2017 | - Ability of relaxation  (faster and deeper) | - Time Interval to Relaxation (TItR); - Hospital Anxiety and Depression Scale (HADS): HADS - Anxiety (A); HADS - Depression (D). | Pre-post comparison | N/R |
| Järvelä et al., 2019 | - Empathy (level and facility to evoke); - Respiration and EEG synchrony | - Values in the VR log files related to the working of the virtual environment; - Respiration data records (aura scale factor, moments when breathing bar waves were sent); - EEG; - Self-reported data about empathy. | 8 conditions: 4 solo scenarios (meditation environment scenarios where the avatar of the other participant was not active); 4 dyadic scenarios (where the other participant’s avatar was active) | Alpha (8-13 Hz) and theta (4-6 Hz) power band changes |
| Kosunen et al., 2016 | - Meditation Depth; - Sense of presence | - Meditation Depth Questionnaire (MEDEQ); - ITC-Sense of Presence Inventory. | Control Condition (meditation exercise on a computer screen without a head- mounted display or NFB); HMD without NFB; Both HMD and NFB. | Alpha and theta-band powers |
| Lu et al., 2022 | - Alpha band power reduction as a NFB index of the users’ attention | - EEG | NFB plus VR vs. Sham NFB plus VR | Alpha power band reduction in parietooccipital regions of the brain |
| Orakpo et al., 2021 | - Sustaining pain relief; - Improving ADL, anxiety, depression and quality of sleep | - Wong-Baker Pain Scale; - Subjective Unit of Distress (SUD); - Participant reports. | Pre-post comparison | Not reported |
| Orakpo et al., 2022 | - Improve centralized pain | - Wong-Baker Pain Scale; - SUD; - Insomnia Severity Index (ISI); - Self-reported levels of pain and severity of insomnia. | Pre-post comparison | VR-NFB therapy at infra-low frequency (trained at the T4 - P4, right parietal) |
| Rolbiecki et al., 2023 | - Cancer-related pain; - Feasibility and acceptability | - Feasibility as measured by Study Retention (i.e., number of patients who completed the entire VR experience vs. the number who started and stopped) and Study Adherence (i.e., number of patients who complied with the VR and data-collection procedures); - Edmonton Symptom Assessment System revised version (ESAS-r); - REDCap (cancer history); - Patient pain rate (from 0 to 10); - Patient Perception of Acceptability brief qualitative follow-up interviews. | Pre-post comparison | N/R |
| Tarrant et al., 2022 | - Improving mood states; - Feasibility | - Brunel Mood Scale | EG (VR plus NFB meditation experience) vs. CG (standard guided audio-only meditation) | Keep the high beta activity below the threshold (indicating a state of inner calm) |
| Yan et al., 2008 | - Strengthening attention; - Feasibility | - Integrated Visual and Auditory (IVA) - Continuous Performance Test (CPT): Response Control Quotient (RCQ); Attention Control Quotient (ACQ). | Pre-post comparison | - Theta/Beta - SMR |
| Yu et al., 2023 | - Ability to down-regulate negative emotions; - System usability | - Emotion Regulation Questionnaire (ERQ); - Difficulties in Emotion Regulation Scale (DERS-16); - Rosenberg Self-Esteem Scale (RSE); - Hospital Anxiety and Depression (HADS); - System Usability Scale (SUS). | Pre-post comparison | N/R |
| Zhang et al., 2019 | - Attention training (accuracy of multitasking and reaction time) | - Game performance recordings | EG (NFB plus VR in participants with high-stress level, some of them with generalized anxiety disorder) vs. CG (NFB plus VR in participants with low-level stress) | N/R |
| Abdessalem and Frasson, 2017 | - Emotional changes (excitement and frustration) | - Neural Agent measurements of excitement and frustration, through EEG | Pre-post comparison | N/R |
| Abdessalem et al., 2021 | - Reduction of negative emotions; - Improving memory and attention performance | - Attention and memory exercises (mean performance); - EEG. | 3 immersive environments: Virtual Reality Train; Adaptive Music Therapy; Intelligent Savannah Therapy | N/R |
| Al-shammari et al., 2022 | - Attention improvement (alpha efficiency) | - EEG; - Performance analysis. | Pre-post comparison | Alpha ratio |
| Berger et al ., 2022 | - SMR (associated with state of relaxation and focus); - Theta and Beta power | - EEG; - Subjective questionnaires on motivation, subjective feeling of presence and flow, on usage of technology and cybersickness. | 3D condition vs. 2D condition (sham group) | SMR |
| Gruzelier et al., 2010 | - Creative acting performance | - EEG; - Acting Performance Scale; - Flow State Scale (FSS); - Presence-in-Performance. | Two training groups (ReaCTor group and Screen group); CG  (no training group) | SMR |
| Kober et al., 2016 | - Mood and Motivation; - NFB Training performance; - User experience | - Visual Analogue Scale (VAS); - Questionnaire on Current Motivation; - Rating of perceived control over visual feedback and subjective level of concentration during NFB training (scale from 0 - no control or poor concentration, to 10 - high control, very good concentration); - Simulator Sickness Questionnaire (SSQ). | CG (2D NFB training) vs. EG (3D VR based NFB) | - Increase SMR Upper Alpha or decrease Theta/Beta ratio |
| Tarrant and Cope, 2018 | - Gamma frontal asymmetry; - Positive changes in mood states | - Positive and Negative Affect Schedule (PANAS); - State-Trait-Cheerfulness-Inventory<30> (STCI-S<30>); - EEG. | Pre-post comparison | Gamma frontal asymmetry |

Legend: N/R – Not reported/unclear; CG – Control group; EG – Experimental group; HMD – Head-Mounted Display; 3D NFB refers to NFB with VR; and NFB 2D refers to conventional NFB (on screen)

**Supplementary table 6.** Virtual Reality and game description

| **Author(s) and date** | **Program name and VR scenarios brief description** |
| --- | --- |
| Benavides et al., 2022 | The VR relaxation session was facilitated by NFB, during which participants underwent learning and practice of relaxation techniques guided by a specialist in virtual meditation. |
| Benedetti et al., 2014 | The training program was structured into two sessions, each comprising a recording and a test phase. The cognitive training was designed to progressively increase in difficulty. At the first level, participants were required to perform a single active movement with the cube in all trials. At the second level, two movements were requested. This sequence of levels is repeated, with the number of movements increasing with each subsequent level. |
| Berger and Davelaar, 2018 | The Stroop task was administered at the beginning and end of each session, with instructions displayed on-screen and two practice blocks following the instructions. Participants received a sound if they did not respond within one second. The NFB training consisted of seven blocks, and each time alpha levels exceeded the threshold, a point was awarded, causing the object to levitate. Between each block, participants were allotted a one-minute break. |
| Chen et al., 2007 | A car racing game is presented in a three-dimensional VR environment. EEG activity was monitored at one-second intervals, with a fluctuating EEG component power value corresponding to the speed or virtual car position  There were two cars: one was controlled by the player, and the other by a computer or another player. The program was divided into four sections: device initialization, EEG signal acquisition and filtering, SMR band power value analysis, and result output. The calculation of SMR band power value served as a link between the VR program and the EEG signal. |
| Cho et al., 2004 | A virtual classroom was created, and participants were able to explore using a HMD. Participants completed a continuous performance task (CPT) before and after the training session to assess attention and response ability. In the CPT, they responded to target stimuli with intervals between stimuli and an exposure time of 250 milliseconds. The task measured hits, reaction times, perceptual sensitivity, omission and commission errors, and response bias. |
| Guedj et al., 2023 | The protocol's central component consisted of sessions conducted within a VR cave. In addition to NFB training, sustained attention (Go/No Go task) and calculation tasks were performed to examine alterations in the TBR. |
| Gu and Frasson, 2017 | Virtual Sophrologist The immersive environments included a seaside setting, a Japanese garden, and a waterfall. A total of 93 sophrology instructions were presented (including instructions to relax and to wake up). The Meditation Score was computed every 16.7 milliseconds from the EEG and translated into one of three kinds of feedback by decision rules (e.g., if the meditation score was less than 0.3333, the user received the feedback "I need to take a long and deep breath."). |
| Järvelä et al., 2019 | DYNECOM This system introduced social dynamics into the environment by enabling multiple users to share the same VR space simultaneously (the presence of another user was represented by an avatar - dyadic). The immersive environment consisted of a relaxed social situation around a shared activity, with a relaxed background related to nature. The meditation environment consisted of six stone statues situated in a ring on a small shrine-like platform. Participants were seated during the experiment, and depending on the test condition, the bridge, the scene lights, and the aura-like ring surrounding active statues exhibited various visual effects or cues. These visual effects or cues were designed to inform the user of their current state. The program included eight distinct meditation environments, four of which were solo scenarios and four were dyadic scenarios in which the other participant's avatar was active. The enhancement of interoception and the deep empathetic processes involved in compassion meditation was achieved through the integration of real-time visualizations of breathing rates and the level of motivation, as assessed by EEG frontal asymmetry and the dyadic synchrony of those signals between the two users. |
| Kosunen et al., 2016 | RelaWorld In the virtual environment, the user was seated on a stone platform oriented towards a representation of the sea. The program offered two distinct meditation practices: body scan and focused attention. The focused attention practice used five focus objects, which were presented floating in front of the user, with one object highlighted. As the practice progressed, the meditative performance was reflected in the user's vertical movement and in the varying opacity of an energy bubble that encircled the user. |
| Lu et al., 2022 | The experiment was conducted using the Multi-Target Tracking (MOT) paradigm, which requires significant attention resources. In the first phase of each trial, 10 spheres appeared randomly in the virtual environment. They turned red for one second and then started moving at a constant speed. If the sphere hit the wall, it would bounce and continue moving. Participants were instructed to track the spheres for six seconds, after which they were required to use the hand controller to select the correct sphere. The correct target sphere would reappear once all three spheres had been selected. |
| Orakpo et al., 2021 | Not mentioned |
| Orakpo et al., 2022 | The VR-NFB involved several games and videos, which served to provide an immersive distraction for the patient. |
| Rolbiecki et al., 2023 | The guided meditations were conducted in natural VR scenes (e.g., waterfall, beach, snowy day, etc.) with calming music and soothing sounds of nature. Users could see their EEG data displayed via a small light ball. If the user became distracted or anxious, the EEG data would pause the experience and change the colors of the scene. |
| Tarrant et al., 2022 | The goal of the game was to keep the high beta activity below the threshold, indicating a state of inner calm. The user's brainwave activity appeared at the bottom of the VR screen, represented by a glowing firefly. When the firefly dropped below the threshold (indicating thinking, analyzing, or stressing) for 5 consecutive seconds, the screen was colored with a red overlay, indicating to the user that their EEG patterns were shifting away from what is typically associated with a state of mental relaxation. |
| Yan et al., 2008 | Three spaceships were flying with one of them being controlled by the participant's brain waves. If the detected "command" signal was within the EEG data threshold, the middle spaceship would accelerate; otherwise, it would maintain its current speed. |
| Yu et al., 2023 | Emo-regulator In this system, the presentation of negative VR scenarios to subjects was used to induce negative emotional responses, which were then regulated by cognitive reappraisal. The VR scenarios changed every four seconds to match the user's emotions. When the participant was happy, the round of regulation ended and a new one began. |
| Zhang et al., 2019 | This game was a fusion of racing games and balloon shooting games. It required players to control a vehicle while reacting to the appearance of balloons with characters. The system checked the game status every 10 seconds to adjust the car speed and balloon generation. Scoring was based on car speed, reaction time, and accuracy. |
| Abdessalem and Frasson, 2017 | AmbuRun This game was based on an ambulance transporting a sick person and the player takes control of the ambulance and tries to get to the hospital with as little damage as possible to save the person. The player had to avoid cars, buses and the road to get there without damage. If the player hits a car, the health of the person in the ambulance decreases, but if the player hits a bus or truck, the person dies, and the user has to try again. As the difficulty increases, the frequency of cars, trucks, and buses increases. |
| Abdessalem et al., 2021 | The program consists of VR environments designed to improve the emotional well-being and cognitive performance of people with SCD. The system used three different therapeutic approaches with different levels of adaptive intelligence: Virtual Reality Train (VR environment had no adaptive intelligence, and participants were able to relax while experiencing a train ride); Adaptive Music Therapy (adaptive therapeutic music, where the music selection is adjusted according to the emotional responses of the participants, monitored in real time via EEG); and Intelligent Savannah Therapy (VR environment designed to simulate a savannah environment, incorporating adaptive intelligence to modify the characteristics of the environment based on the emotions detected in the participants). |
| Al-shammari et al., 2022 | The goal of the system was to improve the patient's attention as evidenced by alpha waves, which are associated with concentration and memory. This program consists of a VR intervention that uses EEG signals to improve attention in ADHD patients and integrates the following components: analysis of the brain's electrical signals; an artificial neural network that uses deep learning techniques to process the EEG data; this network can adapt the images and stimuli shown to the patients based on how they react emotionally and how much attention they pay. |
| Berger et al ., 2022 | In this system, participants had to regulate the movement of either 3D or 2D target objects in a VR scenario during an NF training session, with either real or sham feedback. The 3D condition was based on a well-lit forest environment. Users viewed the environment from a first-person perspective with a green ball in front of them. This ball rolled along a predefined path, collecting light blue floating cubes that marked the path. The ball's movement was based on EEG power, which was individually adjusted for each participant based on an initial baseline run. The ball stopped moving when the signal fell below this threshold and turned red when beta or theta levels exceeded the established limits, indicating excessive artifacts. |
| Gruzelier et al., 2010 | ReaCTor The ReaCTor system (a CAVE type virtual reality environment) involved participants in an immersive representation of a theater auditorium. The participants were surrounded by four screens. When a threshold (EEG data) was exceeded, a "GO" signal was sent to the VR computer, which slowly increased or decreased the lighting level in the auditorium. Simultaneous reductions in theta and beta were induced by reducing intrusive noise from the audience. Participants earned points by successfully increasing activity in the SMR band while avoiding increases in the theta and high beta bands. |
| Kober et al., 2016 | On the 3D display, patients saw a semi-transparent 3D model of a human body created with Unity3D, with the appearance of the organs (brain, heart, vascular system) changing based on the neurofeedback results. A light bulb in the scene brightened or dimmed depending on the success of the EEG modulation, while the brain model turned green or red. In addition, a small moving bar and a reward counter in the lower left corner indicated progress. |
| Tarrant and Cope, 2018 | The experience features a waterfall scene accompanied by soft piano and violin music. Approximately 20 seconds into the session, a female voice begins to guide the viewer through a positivity meditation, encouraging them to recall a moment of gratitude, appreciation, or happiness. As the gamma asymmetry shifts to the left and remains above the threshold, the VR camera gradually moves toward the waterfall and then back. If the gamma asymmetry falls below the threshold at any point, the scene pauses, the screen turns red, and the voice prompts the user to focus on a positive emotional state. The experience lasted between four and five minutes, depending on the number of pauses and restarts. |
